# Genomic Analyses of Transport Proteins in * Ralstonia metallidurans*

**DOI:** 10.1002/cfg.454

**Published:** 2005

**Authors:** Torsten von Rozycki, Dietrich H. Nies, Milton H. Saier

**Affiliations:** 1 Division of Biological Sciences University of California at San Diego La Jolla CA 92093-0116 USA; 2 Molekulare Mikrobiologie Institut für Mikrobiologie Martin-Luther-Universität Halle-Wittenberg Halle 06099 Germany

## Abstract

*Ralstonia (Wautersia, Cupriavidus) metallidurans* (Rme) is better able to withstand high concentrations of heavy metals than any other well-studied organism. This fact renders it a potential agent of bioremediation as well as an ideal model organism for understanding metal resistance phenotypes. We have analysed the genome of Rme for genes encoding homologues of established and putative transport proteins; 13% of all genes in Rme encode such homologues. Nearly one-third of the transporters identified (32%) appear to function in inorganic ion transport with three-quarters of these acting on cations. Transporters specific for amino acids outnumber sugar transporters nearly 3 : 1, and this fact plus the large number of uptake systems for organic acids indicates the heterotrophic preferences of these bacteria. Putative drug efflux pumps comprise 10% of the encoded transporters, but numerous efflux pumps for heavy metals, metabolites and macromolecules were also identified. The results presented should facilitate genetic manipulation and mechanistic studies of transport in this remarkable bacterium.


**Comparative and Functional Genomics**
*Comp Funct Genom* 2005; **6**: 17–56.
Published online in Wiley InterScience (www.interscience.wiley.com). **DOI:** 10.1002/cfg.454

**Research Paper**

### Genomic analyses of transport proteins in *Ralstonia metallidurans*

Torsten von Rozycki[1], Dietrich H. Nies[2] and Milton H. Saier Jr[1]*
[1] *Division of Biological Sciences, University of California at San Diego, La Jolla, CA 92093-0116, USA*
[2] *Molekulare Mikrobiologie, Institut für Mikrobiologie, Martin-Luther-Universität Halle-Wittenberg, 06099 Halle, Germany*

**Correspondence to: Milton H. Saier Jr, Division of Biological Sciences, University of California at San Diego, La Jolla, CA 92093-0116, USA. E-mail: msaier@ucsd.edu*

Received: 14 May 2004
Revised: 14 December 2004
Accepted: 15 December 2004

### Abstract

***Ralstonia (Wautersia, Cupriavidus) metallidurans* (Rme) is better able to withstand high concentrations of heavy metals than any other well-studied organism. This fact renders it a potential agent of bioremediation as well as an ideal model organism for understanding metal resistance phenotypes. We have analysed the genome of Rme for genes encoding homologues of established and putative transport proteins; 13% of all genes in Rme encode such homologues. Nearly one-third of the transporters identified (32%) appear to function in inorganic ion transport with three-quarters of these acting on cations. Transporters specific for amino acids outnumber sugar transporters nearly 3 : 1, and this fact plus the large number of uptake systems for organic acids indicates the heterotrophic preferences of these bacteria. Putative drug efflux pumps comprise 10% of the encoded transporters, but numerous efflux pumps for heavy metals, metabolites and macromolecules were also identified. The results presented should facilitate genetic manipulation and mechanistic studies of transport in this remarkable bacterium. Copyright © 2005 John Wiley & Sons, Ltd.**

**Keywords:** **bioinformatics; transport proteins; comparative genomics**

### Introduction

*Ralstonia metallidurans* (Rme; previously *Alcaligenes eutrophus,* renamed in 2004 *Wautersia metallidurans* and then *Cupriavidus metallidurans*; Goris *et al*., 2001; Vandamme and Coenye, 2004; Vaneechoutte *et al*., 2004), is a Gram-negative facultative chemolithoautotrophic $\beta$-proteobacterium. It was first identified in 1976, when it was isolated from industrial sediments, soils and wastes that were polluted with high concentrations of various heavy metals, such as cobalt, zinc, nickel and cadmium (Mergeay *et al*., 1985). The concentrations of these metals that can exist in the habitats of Rme greatly exceed the values that are lethal to almost any other living organisms. Rme is related to the important plant pathogen *Ralstonia solanacearum* (Boucher *et al*., 2001), which is resistant to a wide variety of drugs and toxic compounds. The complete genome sequence of the latter organism is available (Salanoubat *et al*., 2002).

The properties of Rme render it potentially important for purposes of bioremediation, such as for the degradation of aromatic compounds and xenobiotics, even in the presence of heavy metals as additional pollutants. Rme is also able to synthesize polyhydroxyalkalonates (PHAs), which accumulate as carbon and energy sources and might be useful for the development of biodegradable plastics. The extraordinary heavy metal resistance of Rme and its ability to accumulate these metals on its surface make it a candidate for a variety of clean-up purposes (Legatzki *et al*., 2003a; Mergeay *et al*., 2003; Nies, 2003).

Two low copy number plasmids, pMOL30 (238 kb; Mergeay *et al*., 1985) and pMOL28 (180 kb; Taghavi *et al*., 1997), that are stably carried by Rme strain CH34, are primary determinants of the remarkable heavy metal resistance characteristic of Rme (Legatzki *et al*., 2003a,b). Both are self-transferable at low frequencies, potentially offering a new approach for inserting resistance genes into other organisms. Rme lacks the RecBCD

Copyright © 2005 John Wiley & Sons, Ltd.

pathway for DNA degradation — a property that allows it to serve as an acceptor for foreign resistance genes. The fact that specific transport systems responsible for the uptake and export of various metabolites and heavy metals (Andres *et al.*, 2000; Borremans *et al.*, 2001; Goris *et al.*, 2001; Juhnke *et al.*, 2002; Mergeay *et al.*, 2003; Nies, 2003; Roux *et al.*, 2001) have been better characterized in Rme than in any other bacterium (Nies, 2003), renders Rme a model organism for basic research on metal resistance and homeostasis.

It has been suggested that the resistance of Rme to heavy metals and toxic compounds results from multiple layers of efflux pumps with overlapping substrate specificities (Juhnke *et al.*, 2002; Nies, 2003; Silver, 2003). However, comprehensive genome analyses of the transporters in Rme are still lacking. In this paper we correct this deficiency, reporting bioinformatic studies of all recognizable transporters encoded within the genome of Rme.

### Computer methods

The protein sequences of Rme were extracted from the JGI database and downloaded for all of the analyses reported here. The sequencing work done at JGI (**http://genome.jgi-psf.org/draft_microbes/ralme/ralme.home.html**) and the annotation project performed by the CH34 annotation consortium (**http://genome.ornl.gov/microbial/rmet/**) formed the basis of this work and are acknowledged at this point. Since the names of the CH34 genes have changed many times in the past, as has the name of the organism, cross-reference tables are supplied as supplementary material (**http://bionomie.mikrobiologie.uni-halle.de/SupMat/SupplMat.htm**). Computer-aided searches were conducted to retrieve all proteins encoded within the genome that are recognizably homologous to transport system constituents included in the Transporter Classification Database (TCDB; Busch and Saier 2002; Tran *et al.*, 2003). Briefly, all proteins encoded within the genome were blasted in an automated manner (using BLASTP) against TCDB. Additional databases used for protein functional analysis were the non-redundant SWISSPROT and TrEMBL protein sequence databases. Several protein pattern databases (conserved domain databases at NCBI and Pfam) were also used. Charge bias analyses of membrane protein topology were performed using the TMHMM (Krogh *et al.*, 2001) and WHAT (Zhai and Saier, 2001) programs.

### Results and discussion

### Topological predictions for membrane transporter homologues

The proteome of Rme was analysed for topological predictions; 59% (4072) of the 6985 proteins identified have no predicted TMSs, while 21% (1434) have only one putative TMS. While most of the former proteins are likely to be cytoplasmic, many of the latter will undoubtedly prove to be periplasmic and outer membrane proteins; 8% (580) have two or three TMSs, 5% (320) have four to six TMSs, and 3% each (196 and 223) have seven to 10 and $>10$ TMSs, respectively. Relative to most other prokaryotes analysed, Rme has increased proportions of integral membrane proteins of all topological types (Paulsen *et al.*, 2000).

All putative transport protein constituents recognized in the proteome of Rme were similarly analysed for topology; 932 putative transporter proteins (13%) were recognized in the proteome of Rme. This percentage is higher than observed for most other organisms with fully sequenced genomes (Paulsen *et al.*, 2000). About 24% (227) of these proteins may be cytoplasmic, as they exhibit no putative TMSs. All others are potential integral membrane constituents. Of these, 21% (196) are predicted to have one TMS, 9% (88) have two or three TMSs, 16% (146) have four to six TMSs, 10% (94) have seven to 10 TMSs, and 19% (179) have $\geqslant 11$ TMSs. Many of the one-TMS proteins displayed typical leader sequences at their respective amino-termini and may be secreted via the Sec and Tat export systems (see below). They may be receptors for ABC-, TRAP-T- and TTT-type transport systems (see below). Since transporter families include proteins that are almost always concerned exclusively with transport (Saier, 2003), it is probable that nearly all of these proteins function in transmembrane transport.

Copyright © 2005 John Wiley & Sons, Ltd. 

### Classes of transporters found in *R. metallidurans*

According to the transporter classification (TC) system, transporters are classified into five well-defined categories (classes 1–5) and two poorly defined categories (classes 8 and 9). The well-defined categories are; (a) channels; (b) secondary carriers; (c) primary active carriers; (d) group translocators; and (e) transmembrane electron flow carriers (Busch and Saier, 2002; Saier, 2000). The less well-defined proteins include (8) auxiliary transport proteins and (9) transporters or putative transporters of unknown mechanism of action or function (Saier, 2000).

Table 1 summarizes the distribution of the 932 transporter protein constituents from Rme in each of the major TC categories and also provides a breakdown of these proteins found in the various TC subclasses; 123 channel proteins, most of them outer membrane porins, were identified. However, the majority of defined transport proteins found are secondary carriers (304) and constituents of primary active transporters (343).

Only one phosphoenolpyruvate-dependent, sugar transporting phosphotransferase system (PTS) permease, which catalyses group translocation of hexoses, was found. Further, only 10 transmembrane electron flow system constituents were identified. This latter fact may in part reflect the limited representation of transmembrane electron flow carriers in the Transporter Classification Database (TCDB). Thirty-one auxiliary proteins of TC class 8 and 65 putative transporters of TC class 9 were identified (Table 1). The probable functional identities of the individual proteins will be discussed below.

### Classes of substrates transported

Table 2 summarizes the numbers of transporter proteins concerned with the transport of various types of substrates; 300 proteins are putative transport protein homologues concerned with the uptake or efflux of inorganic ions, and nearly three-quarters of them are concerned with inorganic cation transport. This observation undoubtedly relates to the remarkable heavy metal resistance of Rme.

Forty-one systems specific for sugars and their derivatives and 110 systems specific for amino acids and their derivatives were identified. These facts suggest that amino acid metabolism may be more important to Rme than sugar metabolism for heterotrophic growth. This substrate preference of Rme has been observed before (Mergeay *et al.*, 1985). Rme has 142 transport protein homologues putatively concerned with carboxylate transport, which also agrees with the substrate spectrum of this bacterium (Mergeay *et al.*, 1985). This fact, together with the greater number of secondary carriers relative to primary active transporters, points to a strong metabolic dependency on respiration rather than fermentation. Ninety-one

**Table 1.** Categories of recognized transport proteins found in *Ralstonia metallidurans*

| TC class | No. of transporters (%) | TC subclass | No. of transporters (%) |
|---|---|---|---|
| 1 Channels | 123 (13) | 1.A. $\alpha$-Type channel-forming proteins and peptides | 27 (3) |
| | | 1.B. Outer membrane porins (ß-structure) | 94 (10) |
| | | 1.C. Pore-forming toxins (proteins and peptides) | 1 (0.1) |
| | | 1.E. Holins | 1 (0.1) |
| 2 Secondary carriers | 304 (33) | 2.A. Carrier-type facilitators | 299 (32) |
| | | 2.C. Ion-gradient-driven energizers | 5 (1) |
| 3 Primary transporters | 343 (37) | 3.A. P-P bond hydrolysis-driven transporters | 290 (31) |
| | | 3.B. Decarboxylation-driven active transporters | 2 (0.2) |
| | | 3.D. Oxidoreduction-driven active transporters | 51 (5) |
| 4 Group translocators (PTS) | 2 (0.2) | 4.A. Phosphotransferase systems | 2 (0.2) |
| 5 Transmembrane electron carriers | 10 (1) | 5.A. Transmembrane electron transfer carriers | 10 (1) |
| 8 Auxiliary transport proteins | 31 (3) | 8.A. Auxiliary transport proteins | 31 (3) |
| 9 Poorly-defined systems | 65 (7) | 9.A. Transporters of unknown classification | 10 (1) |
| | | 9.B. Putative uncharacterized transporters | 55 (6) |
| Unclassified | 54 (6) | Unclassified | 54 (6) |
| Total number | 932 (100) | | 932 (100) |

Copyright © 2005 John Wiley & Sons, Ltd. 

**Table 2.** Breakdown of transport proteins according to predicted substrate types in *Ralstonia metallidurans*

| Substrate class | No. of transporters (%) | Substrate subclass | No. of transporters (%) |
|---|---|---|---|
| 1 Inorganic compounds | 300 (32) | Cations | 221 (24) |
| | | Anions | 78 (8) |
| | | $H_2O$ | 1 (0.1) |
| 2 Organic compounds | 400 (43) | Sugars/sugar metabolites | 41 (4) |
| | | Amino acids/polyamines | 110 (12) |
| | | Mono-, di-, tricarboxylates | |
| | | Fatty acids | 142 (15) |
| | | Drugs/toxic compounds | 91 (10) |
| | | Nucleotides/nucleosides | 4 (0.4) |
| | | Aromatics | 13 (1) |
| 3 Macromolecules | 102 (11) | Lipoproteins/proteins | 75 (8) |
| | | Lipopolysaccharides/polysaccharides | 20 (2) |
| | | DNA | 5 (0.5) |
| | | Lipids | 1 (0.1) |
| 4 Miscellaneous/unknown | 130 (14) | Miscellaneous | 15 (2) |
| | | Unknown | 115 (12) |
| Total | 932 (100) | | 932 (100) |

proteins are predicted to be concerned with transport of drugs and hydrophobic substances, while 130 proteins fall into the miscellaneous/unknown category.

### Global analysis of transporters in Rme and their family associations

Table 3 summarizes the results of our detailed analyses of transporters found in Rme. On the left, the family TC number, the name of the family and its standard abbreviation can be found (columns 1–3). Column 4 presents the types of substrates known to be transported by members of the respective family. Column 5 gives the number of family members identified in Rme, while column 6 presents the gene designation used in the draft version (02jul03) of the Rme genome analysed here. A full version of this table that contains all of the various names of the CH34 genes is provided as supplementary material (**http://bionomie.mikrobiologie.uni-halle.de/SupMat/Roz_05/Table 3.htm**). Column 7 gives the protein size in number of amino acyl residues, and column 8 provides an estimate of the number of putative transmembrane spanning regions (TMSs) for each protein. The TC number of the protein in TCDB that shows greatest similarity to the Rme ORF under consideration is presented in column 9. Finally, column 10 presents the level of confidence for the functional assignment (1 = sure, 2 = probable, 3 = uncertain or unknown).

### Channels

In category 1A ($\alpha$-type channels), Rme possesses two members of the VIC family (1.A.1), both probably $K^+$ channels. Two members of the MIP family of aqua/glycerol porins are also present. Four putative chloride channels (ClC family) were found, as well as one CytB homologue. This last system may function primarily in transmembrane electron flow, but no bacterial member of this family has been characterized (Kimball and Saier, 2002).

MscL (1.A.22), MscS (1.A.23) and MIT (1.A.35) families are all well represented with one, nine and four members, respectively. All four MIT family members are probably divalent cation transporters, while the MscL and MscS proteins are most likely non-specific channels for protection against osmotic stress (Busch and Saier, 2002; Nottebrock *et al.*, 2003; Pivetti *et al.*, 2003). Rme exhibits two paralogues within the hsp70 family of chaperone proteins, some of which have been shown to be capable of forming transmembrane channels (Arispe and De Maio, 2000). No other channel-type proteins of TC class 1.A could be recognized.

A tremendous number of putative outer membrane porins were identified. For example, just within the general bacterial porin (GBP) family (1.B.1), 29 paralogues were found. Most of these proteins are of 300–400 amino acids in length and probably consist largely of $\beta$-structure. A trimeric

Copyright © 2005 John Wiley & Sons, Ltd. 

**Table 3.** Putative transport proteins identified in *Ralstonia metallidurans*[a]

| Family (1) | Family (2) | Abbreviation (3) | Typical substrates (4) | Total # (5) | Gene (02jul03) (6) | Length (#aas) (7) | # TMSs (8) | Nearest homologue in TCDB (9) | Evidence (10) |
|---|---|---|---|---|---|---|---|---|---|
| 1.A. $\alpha$-Type channel-forming proteins and peptides | | | | | | | | | |
| 1.A.1 | Voltage-gated ion channel | VIC | $Na^+$, $K^+$, $Ca^{2+}$, multiple cations | | Contig372gene5732 | 307 | 5 | 1.A.1.2.3(1) | 3 |
| | | | | 2 | Contig373gene6187 | 229 | 7 | 1.A.1.13.1(1) | 3 |
| 1.A.8 | Major intrinsic protein | MIP | $H_2O$, glycerol, urea, polyols, $NH_3$, $CO_2$ | | Contig375gene7720 | 250 | 6 | 1.A.8.13.1(1) | 3 |
| | | | | 2 | Contig375gene8643 | 234 | 6 | 1.A.8.3.1(1) | 2 |
| 1.A.11 | Chloride channel | ClC | $Cl^-$, anions | | Contig365gene3384 | 376 | 4 | 1.A.11.6.1(1) | 3 |
| | | | | | Contig365gene3245 | 657 | 12 | 1.A.11.6.1(1) | 3 |
| | | | | | Contig352gene1115 | 560 | 8 | 1.A.11.6.1(1) | 3 |
| | | | | 4 | Contig367gene3837 | 522 | 10 | 1.A.11.6.1(1) | 3 |
| 1.A.20 | gp91$_{phox}$ Phagocyte NADPH oxidase-associated cytochrome b558 | CytB | $H^+$ | 1 | Contig363gene2857 | 447 | 6 | 1.A.20.6.1(1) | 2 |
| 1.A.22 | Large conductance mechanosensitive ion channel | MscL | Proteins, ions (slightly cation selective) | 1 | Contig367gene3927 | 144 | 2 | 1.A.22.1.3(1) | 2 |
| 1.A.23 | Small conductance mechanosensitive ion channel | MscS | Ions (slight anion selectivity) | | Contig350gene938 | 456 | 6 | 1.A.23.1.1(1) | 2 |
| | | | | | Contig373gene6759 | 357 | 4 | 1.A.23.1.1(1) | 3 |
| | | | | | Contig375gene8191 | 275 | 3 | 1.A.23.1.1(1) | 3 |
| | | | | | Contig375gene8051 | 832 | 9 | 1.A.23.1.1(1) | 2 |
| | | | | | Contig375gene7869 | 288 | 4 | 1.A.23.2.1(1) | 2 |
| | | | | | Contig372gene5633 | 284 | 4 | 1.A.23.2.1(1) | 2 |
| | | | | | Contig371gene5100 | 771 | 13 | 1.A.23.3.1(1) | 3 |
| | | | | | Contig358gene1757 | 570 | 5 | 1.A.23.4.1 | 3 |
| | | | | 9 | Contig361gene2514 | 447 | 5 | 1.A.23.4.1(1) | 3 |
| 1.A.30 | $H^+$- or $Na^+$-translocating bacterial flagellar motor 1ExbBD outer membrane transport energizer | Mot/Exb-Mot | $H^+$, $Na^+$ | | Contig371gene5340 | 299 | 4 | 1.A.30.1.1(2) | 2 |
| | | | | 2 | Contig371gene5341 | 325 | 1 | 1.A.30.1.1(2) | 2 |
| 1.A.33 | Cation channel-forming heat shock protein-70 | Hsp70 | Ions, polypeptides | | Contig370gene5054 | 621 | 0 | 1.A.33.1.2(1) | 2 |
| | | | | 2 | Contig372gene5852 | 648 | 0 | 1.A.33.1.2(1) | 2 |
| 1.A.35 | CorA metal ion transporter | MIT | Heavy-metal ions, $Mg^{2+}$, $Mn^{2+}$, $Co^{2+}$, $Ni^{2+}$, $Fe^{2+}$, $Al^{3+}$, $Mn^{2+}$ | | Contig363gene2888 | 320 | 2 | 1.A.35.1.2(1) | 2 |

Copyright © 2005 John Wiley & Sons, Ltd.

**Table 3.** Continued

| Family (1) | Family (2) | Abbreviation (3) | Typical substrates (4) | Total # (5) | Gene (02jul03) (6) | Length (#aas) (7) | # TMSs (8) | Nearest homologue in TCDB (9) | Evidence (10) |
|---|---|---|---|---|---|---|---|---|---|
| | | | | | Contig374gene7317 | 383 | 3 | 1.A.35.3.1(1) | 3 |
| | | | | | Contig365gene3224 | 362 | 3 | 1.A.35.3.1(1) | 3 |
| | | | | 4 | Contig367gene3951 | 393 | 2 | 1.A.35.3.1(1) | 3 |
| 1.B. Outer membrane porins (β-structure) | | | | | | | | | |
| 1.B.1 | General bacterial porin | GBP | Ions, small ($M_r$ of <1000 Da) molecules | | Contig358gene1907 | 367 | 1 | 1.B.1.4.1(1) | 2 |
| | | | | | Contig340gene366 | 432 | 3 | 1.B.1.4.1(1) | 3 |
| | | | | | .00Dec2000-Contig485gene1110 | | | 1.B.1.4.1(1) | 3 |
| | | | | | Contig373gene6049 | 381 | 1 | 1.B.1.4.1(1) | 3 |
| | | | | | Contig373gene6801 | 393 | 6 | 1.B.1.4.1(1) | 3 |
| | | | | | Contig374gene7003 | 111 | 1 | 1.B.1.4.1(1) | 2 |
| | | | | | Contig338gene292 | 382 | 1 | 1.B.1.4.1(1) | 3 |
| | | | | | Contig375gene8510 | 352 | 0 | 1.B.1.4.1(1) | 2 |
| | | | | | Contig375gene8258 | 379 | 1 | 1.B.1.4.1(1) | 2 |
| | | | | | Contig375gene8489 | 355 | 3 | 1.B.1.4.1(1) | 2 |
| | | | | | Contig375gene9145 | 353 | 1 | 1.B.1.4.1(1) | 2 |
| | | | | | Contig375gene9285 | 341 | 0 | 1.B.1.4.1(1) | 3 |
| | | | | | Contig354gene1343 | 377 | 1 | 1.B.1.4.1(1) | 2 |
| | | | | | Contig364gene3106 | 371 | 1 | 1.B.1.4.1(1) | 2 |
| | | | | | Contig375gene8681 | 352 | 1 | 1.B.1.4.1(1) | 2 |
| | | | | | Contig373gene6282 | 386 | 1 | 1.B.1.4.1(1) | 2 |
| | | | | | Contig371gene5423 | 391 | 1 | 1.B.1.4.1(1) | 2 |
| | | | | | Contig372gene5670 | 387 | 2 | 1.B.1.4.1(1) | 2 |
| | | | | | Contig372gene5912 | 358 | 0 | 1.B.1.4.1(1) | 2 |
| | | | | | Contig372gene5687 | 362 | 0 | 1.B.1.4.1(1) | 2 |
| | | | | | Contig370gene4663 | 361 | 1 | 1.B.1.6.1(1) | 3 |
| | | | | | Contig373gene6196 | 382 | 1 | 1.B.1.6.1(1) | 2 |
| | | | | | Contig357gene1691 | 374 | 1 | 1.B.1.6.1(1) | 2 |
| | | | | | Contig358gene1746 | 354 | 1 | 1.B.1.6.1(1) | 2 |
| | | | | | Contig361gene2442 | 371 | 1 | 1.B.1.6.1(1) | 2 |
| | | | | | Contig373gene6139 | 355 | 3 | 1.B.1.6.1(1) | 2 |
| | | | | | Contig373gene6638 | 371 | 1 | 1.B.1.6.1(1) | 3 |
| | | | | | Contig371gene5227 | 363 | 1 | 1.B.1.6.1(1) | 2 |
| | | | | 29 | Contig369gene4254 | 355 | 3 | 1.B.1.6.1(1) | 2 |
| 1.B.6 | OmpA-OmpF porin | OOP | Ions, small molecules | | Contig370gene4785 | 217 | 0 | 1.B.6.1.1(1) | 2 |
| | | | | | Contig365gene3355 | 643 | 2 | 1.B.6.1.2(1) | 3 |
| | | | | 3 | Contig373gene6115 | 217 | 1 | 1.B.6.1.3(1) | 3 |
| 1.B.9 | FadL outer membrane protein | FadL | Fatty acid, toluene, *m*-xylene and benzyl alcohol | 1 | Contig343gene479 | 464 | 1 | 1.B.9.2.1(1) | 2 |

Copyright © 2005 John Wiley & Sons, Ltd.

| | | | | | | | | | |
|---|---|---|---|---|---|---|---|---|---|
| I.B.11 | Outer membrane fimbrial usher porin | FUP | Protein folding and subunit assembly | | Contig358gene1879 | 761 | 0 | I.B.11.3.1(1) | 2 |
| | | | | | Contig365gene3393 | 854 | 1 | I.B.11.3.1(1) | 2 |
| | | | | 3 | Contig354gene1279 | 850 | 1 | I.B.11.3.1(1) | 2 |
| I.B.12 | Autotransporter | AT | N-terminal protein domains | 1 | Contig365gene3360 | 1741 | 1 | I.B.12.1.3(1) | 2 |
| I.B.14 | Outer membrane receptor | OMR | Iron-siderophore complexes, vitamin $B_{12}$, $Cu^{2+}$, colicin, DNA of various phages | | Contig374gene7240 | 733 | 1 | I.B.14.1.2(1) | 3 |
| | | | | | Contig372gene5928 | 731 | 1 | I.B.14.1.2(1) | 2 |
| | | | | | Contig372gene5930 | 717 | 0 | I.B.14.1.2(1) | 2 |
| | | | | | Contig370gene4894 | 764 | 1 | I.B.14.1.4(1) | 2 |
| | | | | | Contig361gene2288 | 742 | 1 | I.B.14.1.4(1) | 2 |
| | | | | | Contig374gene7149 | 744 | 1 | I.B.14.1.4(1) | 2 |
| | | | | | Contig374gene7151 | 804 | 0 | I.B.14.1.4(1) | 3 |
| | | | | | Contig363gene2909 | 753 | 1 | I.B.14.1.4(1) | 2 |
| | | | | | Contig369gene4344 | 728 | 1 | I.B.14.1.4(1) | 2 |
| | | | | | Contig369gene4384 | 761 | 2 | I.B.14.1.4(1) | 3 |
| | | | | | Contig375gene8531 | 741 | 0 | I.B.14.1.4(1) | 2 |
| | | | | | Contig374gene6949 | 719 | 0 | I.B.14.1.6(1) | 3 |
| | | | | | Contig373gene6356 | 661 | 0 | I.B.14.1.6(1) | 2 |
| | | | | | Contig375gene8072 | 821 | 1 | I.B.14.1.8(1) | 3 |
| | | | | | Contig366gene3585 | 698 | 1 | I.B.14.3.1(1) | 2 |
| | | | | | Contig375gene8595 | 724 | 0 | I.B.14.4.1(1) | 2 |
| | | | | 17 | Contig369gene4334 | 815 | 1 | I.B.14.9.1(1) | 2 |
| I.B.17 | Outer membrane factor | OMF | Heavy metal cations, drugs, oligosaccharides, proteins, etc. | | Contig366gene3474 | 493 | 0 | I.B.17.1.1(1) | 2 |
| | | | | | Contig369gene4234 | 448 | 1 | I.B.17.2.1(1) | 1 |
| | | | | | Contig368gene3997 | 418 | 0 | I.B.17.2.1(1) | 1 |
| | | | | | Contig357gene1641 | 460 | 0 | I.B.17.2.2(1) | 1 |
| | | | | | Contig371gene5461 | 445 | 0 | I.B.17.2.2(1) | 3 |
| | | | | | Contig374gene7266 | 431 | 1 | I.B.17.2.2(1) | 1 |
| | | | | | Contig375gene8615 | 418 | 0 | I.B.17.2.2(1) | 1 |
| | | | | | Contig374gene7202 | 520 | 2 | I.B.17.2.3(1) | 3 |
| | | | | | Contig373gene6079 | 433 | 0 | I.B.17.2.3(1) | 3 |
| | | | | | Contig375gene9177 | 496 | 0 | I.B.17.3.1(1) | 2 |
| | | | | | Contig375gene8190 | 485 | 1 | I.B.17.3.1(1) | 2 |
| | | | | | Contig353gene1195 | 455 | 0 | I.B.17.3.2(1) | 3 |
| | | | | | Contig360gene2100 | 519 | 0 | I.B.17.3.2(1) | 2 |
| | | | | | Contig354gene1322 | 418 | 1 | I.B.17.3.2(1) | 2 |
| | | | | | Contig364gene3065 | 504 | 1 | I.B.17.3.2(1) | 2 |
| | | | | | Contig358gene1809 | 486 | 2 | I.B.17.3.3(1) | 2 |
| | | | | | Contig375gene7574 | 497 | 1 | I.B.17.3.3(1) | 2 |
| | | | | | Contig359gene2067 | 488 | 1 | I.B.17.3.3(1) | 2 |

Copyright © 2005 John Wiley & Sons, Ltd.

**Table 3.** Continued

| Family (1) | Family (2) | Abbreviation (3) | Typical substrates (4) | Total # (5) | Gene (02jul03) (6) | Length (#aas) (7) | # TMSs (8) | Nearest homologue in TCDB (9) | Evidence (10) |
|---|---|---|---|---|---|---|---|---|---|
| | | | | | Contig353gene1181 | 488 | 1 | 1.B.17.3.3(1) | 2 |
| | | | | | Contig373gene6314 | 518 | 1 | 1.B.17.3.3(1) | 2 |
| | | | | | Contig373gene6558 | 511 | 3 | 1.B.17.3.3(1) | 2 |
| | | | | | Contig375gene8564 | 495 | 0 | 1.B.17.3.3(1) | 2 |
| | | | | | Contig358gene1815 | 519 | 0 | 1.B.17.3.4(1) | 2 |
| | | | | | Contig373gene6386 | 589 | 0 | 1.B.17.3.4(1) | 3 |
| | | | | | Contig362gene2648 | 512 | 0 | 1.B.17.3.4(1) | 2 |
| | | | | | Contig353gene1190 | 497 | 1 | 1.B.17.3.5(1) | 2 |
| | | | | | Contig375gene8587 | 476 | 0 | 1.B.17.3.5(1) | 2 |
| | | | | 28 | Contig375gene7766 | 491 | 2 | 1.B.17.3.5(1) | 2 |
| 1.B.18 | Outer membrane auxiliary protein | OMA | Exo- or capsular polysaccharide | | Contig375gene8672 | 606 | 0 | 1.B.18.1.2(1) | 2 |
| | | | | 2 | Contig372gene5594 | 362 | 0 | 1.B.18.3.1(1) | 2 |
| 1.B.19 | Glucose-selective OprB porin | OprB | Ions, small molecules | 1 | Contig359gene1948 | 492 | 1 | 1.B.19.1.1(1) | 3 |
| 1.B.20 | Two-partner secretion | TPS | Proteins | | Contig373gene6550 | 588 | 0 | 1.B.20.1.1(1) | 2 |
| | | | | 2 | Contig371gene5256 | 558 | 1 | 1.B.20.3.1(1) | 3 |
| 1.B.22 | Outer bacterial membrane secretin | Secretin | Proteins | | Contig371gene5305 | 473 | 0 | 1.B.22.1.1(1) | 2 |
| | | | | | Contig365gene3336 | 620 | 1 | 1.B.22.1.2(1) | 3 |
| | | | | | Contig375gene7610 | 783 | 1 | 1.B.22.1.2(1) | 2 |
| | | | | | Contig367gene3787 | 710 | 1 | 1.B.22.2.1(1) | 2 |
| | | | | | Contig375gene9238 | 734 | 1 | 1.B.22.4.1(1) | 2 |
| | | | | 6 | Contig368gene4122 | 600 | 0 | 1.B.22.7.1(1) | 3 |
| 1.B.39 | Bacterial porin, OmpW | OmpW | Methyl viologen and benzyl viologen | | Contig375gene9331 | 286 | 1 | 1.B.39.1.1(1) | 3 |
| | | | | 2 | Contig372gene5565 | 245 | 0 | 1.B.39.1.1(1) | 3 |
| 1.C. Pore-forming toxins (proteins and peptides) | | | | | | | | | |
| 1.C.1 | Channel-forming colicin | Colicin | Ions, small molecules | 1 | Contig353gene1187 | 443 | 1 | 1.C.1.3.1(1) | 3 |
| 1.E. Holins | | | | | | | | | |
| 1.E.14 | LrgA holin | LrgA Holin | $Zn^{2+}$, $Fe^{2+}$ | 1 | Contig372gene5735 | 128 | 3 | 1.E.14.1.1(1) | 3 |
| 2.A. Carrier type facilitators | | | | | | | | | |
| 2.A.1 | Major facilitator superfamily | MFS | Various small molecules | | | | | | |
| | | - SP (1) | Sugars | 1 | Contig373gene6468 | 484 | 12 | 2.A.1.1.15(1) | 3 |
| | | - DHA1 (12 spanner) (2) | Drugs | | Contig374gene7546 | 418 | 12 | 2.A.1.2.4(1) | 3 |
| | | | | | Contig358gene1790 | 411 | 12 | 2.A.1.2.4(1) | 3 |
| | | | | | Contig370gene4856 | 418 | 12 | 2.A.1.2.7(1) | 2 |
| | | | | | Contig375gene9203 | 426 | 11 | 2.A.1.2.7(1) | 2 |
| | | | | | Contig369gene4402 | 634 | 12 | 2.A.1.2.9(1) | 3 |
| | | | | | Contig375gene8690 | 408 | 12 | 2.A.1.2.8(1) | 3 |
| | | | | | Contig371gene5419 | 415 | 12 | 2.A.1.2.8(1) | 3 |

Copyright © 2005 John Wiley & Sons, Ltd.

| | | | | | | | |
|---|---|---|---|---|---|---|---|
| | | | Contig373gene6337 | 404 | 12 | 2.A.1.2.10(1) | 3 |
| | | | Contig373gene6438 | 275 | 6 | 2.A.1.2.10(1) | 3 |
| | | | Contig375gene8780 | 408 | 12 | 2.A.1.2.10(1) | 3 |
| | | | Contig369gene4487 | 426 | 11 | 2.A.1.2.10(1) | 3 |
| | | | Contig373gene6352 | 388 | 12 | 2.A.1.2.14(1) | 2 |
| | | | Contig375gene9023 | 421 | 12 | 2.A.1.2.14(1) | 2 |
| | | | Contig375gene7618 | 400 | 12 | 2.A.1.2.14(1) | 3 |
| | | | Contig375gene7724 | 398 | 12 | 2.A.1.2.18(1) | 3 |
| | | 16 | Contig369gene4338 | 395 | 12 | 2.A.1.2.20(1) | 3 |
| - DHA2 (14 spanner) (3) | Drugs | | Contig360gene2102 | 537 | 14 | 2.A.1.3.2(1) | 2 |
| | | | Contig358gene1921 | 532 | 14 | 2.A.1.3.2(1) | 2 |
| | | | Contig364gene3067 | 519 | 14 | 2.A.1.3.2(1) | 2 |
| | | | Contig366gene3427 | 396 | 12 | 2.A.1.3.5(1) | 3 |
| | | | Contig375gene7796 | 528 | 14 | 2.A.1.3.5(1) | 2 |
| | | | Contig339gene328 | 480 | 14 | 2.A.1.3.11(1) | 2 |
| | | | Contig335gene226 | 548 | 14 | 2.A.1.3.12(1) | 2 |
| | | | Contig373gene6800 | 504 | 14 | 2.A.1.3.17(1) | 3 |
| | | | Contig374gene7112 | 490 | 14 | 2.A.1.3.17(1) | 2 |
| | | | Contig375gene8085 | 473 | 14 | 2.A.1.3.17(1) | 2 |
| | | | Contig373gene6605 | 513 | 14 | 2.A.1.3.17(1) | 2 |
| | | | Contig351gene1053 | 508 | 14 | 2.A.1.3.18(1) | 2 |
| | | | Contig375gene8189 | 524 | 13 | 2.A.1.3.18(1) | 2 |
| | | | Contig375gene8551 | 545 | 14 | 2.A.1.3.18(1) | 2 |
| | | 15 | Contig371gene5277 | 525 | 14 | 2.A.1.3.18(1) | 3 |
| - MHS (6) | Dicarboxylates, tricarboxylates | | Contig373gene6395 | 434 | 12 | 2.A.1.6.1(1) | 2 |
| | | | Contig361gene2390 | 429 | 12 | 2.A.1.6.1(1) | 2 |
| | | | Contig374gene7196 | 476 | 12 | 2.A.1.6.1(1) | 2 |
| | | | Contig375gene8112 | 436 | 12 | 2.A.1.6.1(1) | 2 |
| | | | Contig373gene6395 | 434 | 0 | 2.A.1.6.1(1) | 3 |
| | | | Contig354gene1283 | 262 | 7 | 2.A.1.6.1(1) | 2 |
| | | | Contig372gene5669 | 443 | 12 | 2.A.1.6.1(1) | 2 |
| | | | Contig375gene7621 | 461 | 12 | 2.A.1.6.1(1) | 2 |
| | | | Contig372gene5548 | 437 | 12 | 2.A.1.6.3(1) | 2 |
| | | | Contig352gene1110 | 565 | 0 | 2.A.1.6.4(1) | 3 |
| | | | Contig358gene1834 | 255 | 6 | 2.A.1.6.5(1) | 2 |
| | | | Contig358gene1835 | 300 | 6 | 2.A.1.6.5(1) | 2 |
| | | | Contig352gene1110 | 565 | 12 | 2.A.1.6.5(1) | 2 |
| | | | Contig373gene6129 | 459 | 12 | 2.A.1.6.6(1) | 2 |
| | | 15 | Contig375gene8177 | 439 | 12 | 2.A.1.6.6(1) | 2 |
| - NNP (8) | Nitrate, nitrite | | Contig375gene7921 | 437 | 12 | 2.A.1.8.4(1) | 2 |
| | | | Contig360gene2126 | 427 | 12 | 2.A.1.8.11(1) | 2 |
| | | 3 | Contig360gene2127 | 460 | 12 | 2.A.1.8.11(1) | 2 |
| - OFA (11) | Oxalate, formate | | Contig364gene3127 | 441 | 12 | 2.A.1.11.1(1) | 2 |

Copyright © 2005 John Wiley & Sons, Ltd. 

**Table 3.** Continued

| Family (1) | Family (2) | Abbreviation (3) | Typical substrates (4) | Total # (5) | Gene (02jul03) (6) | Length (#aas) (7) | # TMSs (8) | Nearest homologue in TCDB (9) | Evidence (10) |
|---|---|---|---|---|---|---|---|---|---|
| | | | | | Contig355gene1391 | 1472 | 12 | 2.A.1.11.1(1) | 3 |
| | | | | 3 | Contig357gene1707 | 475 | 12 | 2.A.1.11.1(1) | 3 |
| | | - SHS (12) | Sialate, lactate, pyruvate | 1 | Contig359gene2081 | 397 | 12 | 2.A.1.12.1(1) | 3 |
| | | - ACS (14) | Organic acids | | Contig353gene1192 | 443 | 12 | 2.A.1.14.1(1) | 3 |
| | | | | | Contig375gene9216 | 453 | 12 | 2.A.1.14.1(1) | 2 |
| | | | | | Contig371gene5115 | 433 | 12 | 2.A.1.14.1(1) | 2 |
| | | | | | Contig365gene3217 | 444 | 12 | 2.A.1.14.1(1) | 2 |
| | | | | | Contig365gene3305 | 418 | 12 | 2.A.1.14.2(1) | 3 |
| | | | | | Contig346gene689 | 437 | 12 | 2.A.1.14.3(1) | 2 |
| | | | | | Contig361gene2423 | 441 | 12 | 2.A.1.14.3(1) | 2 |
| | | | | | Contig359gene1940 | 453 | 12 | 2.A.1.14.8(1) | 2 |
| | | | | 9 | Contig375gene8175 | 432 | 12 | 2.A.1.14.8(1) | 3 |
| | | - AAHS(15) | Aromatic acids | | Contig364gene3071 | 413 | 12 | 2.A.1.15.1(1) | 3 |
| | | | | | Contig375gene8062 | 459 | 12 | 2.A.1.15.1(1) | 2 |
| | | | | | Contig371gene5360 | 441 | 12 | 2.A.1.15.1(1) | 2 |
| | | | | | Contig373gene6741 | 395 | 12 | 2.A.1.15.3(1) | 3 |
| | | | | 5 | Contig375gene9515 | 441 | 12 | 2.A.1.15.4(1) | 2 |
| | | - CP (17) | Cyanate | 1 | Contig370gene4875 | 423 | 12 | 2.A.1.17.1(1) | 3 |
| | | - OCT (19) | Organic cations | 1 | Contig373gene6048 | 526 | 12 | 2.A.1.19.4(1) | 2 |
| | | - SET (20) | Sugars | 1 | Contig373gene6742 | 434 | 0 | 2.A.1.20.2(1) | 3 |
| | | - DHA3 (12 spanner) (21) | Drugs | 1 | Contig351gene976 | 493 | 12 | 2.A.1.21.3(1) | 3 |
| | | - VNT (22) | Neurotransmitter | 1 | Contig375gene7913 | 514 | 12 | 2.A.1.22.1(1) | 3 |
| | | - BST (23) | Unknown | 1 | Contig375gene8194 | 436 | 12 | 2.A.1.23.1(1) | 3 |
| | | - PAT (25) | Peptides, AcCoA | 1 | Contig374gene6932 | 466 | 12 | 2.A.1.25.2(1) | 2 |
| | | - UMC-terminal fragment (26) | Unknown | 1 | Contig368gene4202 | 413 | 12 | 2.A.1.26.1(1) | 3 |
| | | - PPP (27) | Phenylpropionate | 1 | Contig369gene4405 | 365 | 11 | 2.A.1.27.1(1) | 2 |
| | | - ADT (30) | Abietane diterpenoid | 1 | Contig372gene5630 | 468 | 12 | 2.A.1.30.1(1) | 3 |
| | | - Nre (31) | $Ni^{2+}$ | 1 | Contig369gene4238 | 408 | 12 | 2.A.1.31.1(1) | 2 |
| | | - Fsr (35) | Fosmidomycin | 1 | Contig375gene7801 | 409 | 12 | 2.A.1.35.1(1) | 2 |
| | | - AtoE (37) | Short chain fatty | | Contig364gene2968 | 467 | 14 | 2.A.1.37.1(1) | 2 |
| | total 83 | | | 2 | Contig375gene9151 | 484 | 14 | 2.A.1.37.1(1) | 2 |
| 2.A.3 | Amino acid-polyamine-organocation | APC | Amino acids, polyamines, choline | | | | | | |
| | | - AAA (1) | Amino acids | | Contig361gene2312 | 493 | 12 | 2.A.3.1.2(1) | 2 |
| | | | | | Contig375gene8013 | 510 | 12 | 2.A.3.1.2(1) | 2 |
| | | | | | Contig375gene8010 | 462 | 12 | 2.A.3.1.3(1) | 2 |

Copyright © 2005 John Wiley & Sons, Ltd.

| | | | | | | | | | |
|---|---|---|---|---|---|---|---|---|---|
| | | | | | Contig373gene6637 | 475 | 12 | 2.A.3.1.9(1) | 2 |
| | | | | 5 | Contig373gene6757 | 474 | 12 | 2.A.3.1.9(1) | 2 |
| | total 6 | - CAT (3) | Cationic amino acids | 1 | Contig374gene7465 | 469 | 14 | 2.A.3.3.1(1) | 2 |
| 2.A.4 | Cation diffusion facilitator | CDF | $Cd^{2+}$, $Co^{2+}$, $Zn^{2+}$ | | Contig375gene8618 | 316 | 5 | 2.A.4.1.1(1) | 1 |
| | | | | | Contig375gene9479 | 337 | 6 | 2.A.4.1.1(1) | 3 |
| | | | | 3 | Contig374gene6900 | 436 | 6 | 2.A.4.1.2(1) | 2 |
| 2.A.5 | Zinc ($Zn^{2+}$)—iron ($Fe^{2+}$) permease | ZIP | | 1 | Contig356gene1473 | 291 | 6 | 2.A.5.4.1(1) | 3 |
| 2.A.6 | Resistance-nodulation-cell division | RND | | | | | | | |
| | | - HME (1) | Heavy metal ions, multiple drugs, oligosaccharides, organic solvents, fatty acids, phospholipids, cholesterol | | Contig369gene4237 | 1076 | 11 | 2.A.6.1.1(1) | 1 |
| | | | | | Contig368gene3999 | 1076 | 12 | 2.A.6.1.1(1) | 1 |
| | | | | | Contig331gene151 | 1043 | 12 | 2.A.6.1.2(1) | 1 |
| | | | | | Contig371gene5463 | 840 | 0 | 2.A.6.1.X | 3 |
| | | | | | Contig371gene5464 | 184 | 5 | 2.A.6.1.X | 1 |
| | | | | | Contig373gene6557 | 1039 | 12 | 2.A.6.1.2(1) | 1 |
| | | | | | Contig373gene6563 | 1045 | 12 | 2.A.6.1.2(1) | 1 |
| | | | | | Contig375gene8617 | 1063 | 12 | 2.A.6.1.2(1) | 1 |
| | | | | | Contig361gene2416 | 1036 | 12 | 2.A.6.1.2(1) | 1 |
| | | | | | Contig375gene8282 | 1023 | 11 | 2.A.6.1.2(1) | 1 |
| | | | | | Contig363gene2863 | 1009 | 7 | 2.A.6.1.2(1) | 1 |
| | | | | | Contig375gene8486 | 691 | 6 | 2.A.6.1.2(1) | 1 |
| | | | | | Contig375gene8119 | 365 | 5 | 2.A.6.1.2(1) | 1 |
| | | | | | Contig373gene6081 | 1055 | 14 | 2.A.6.1.3(1) | 2 |
| | | | | | Contig357gene1642 | 384 | 0 | 2.A.6.1.4(4) | 3 |
| | | | | | Contig369gene4482 | 521 | 1 | 2.A.6.1.4(4) | 2 |
| | | | | 17 | Contig369gene4483 | 1056 | 14 | 2.A.6.1.4(1) | 2 |
| | | - HAE1 (2) | Hydrophobe/amphiphile substrates | | Contig375gene7765 | 1050 | 12 | 2.A.6.2.2(1) | 2 |
| | | | | | Contig369gene4331 | 1044 | 12 | 2.A.6.2.7(1) | 2 |
| | | | | | Contig358gene1808 | 1063 | 12 | 2.A.6.2.9(1) | 2 |
| | | | | | Contig375gene7572 | 1051 | 12 | 2.A.6.2.12(1) | 2 |
| | | | | | Contig375gene7573 | 1100 | 12 | 2.A.6.2.12(1) | 2 |
| | | | | | Contig365gene3373 | 1066 | 0 | 2.A.6.2.12(1) | 2 |
| | | | | | Contig365gene3373 | 1066 | 12 | 2.A.6.2.12(1) | 2 |
| | | | | | Contig353gene1179 | 1065 | 12 | 2.A.6.2.12(1) | 2 |
| | | | | 9 | Contig375gene7759 | 1069 | 12 | 2.A.6.2.12(1) | 2 |
| | | - SecDF (4) | Sec secretory accessory proteins | | Contig372gene5750 | 636 | 5 | 2.A.6.4.1(2) | 2 |
| | | | | 2 | Contig372gene5751 | 324 | 6 | 2.A.6.4.1(2) | 2 |
| | | - HAE2 (5) | Hydrophobe/amphiphile substrates | 1 | Contig364gene3205 | 858 | 9 | 2.A.6.5.1(1) | 3 |

Copyright © 2005 John Wiley & Sons, Ltd.

**Table 3.** Continued

| Family (1) | Family (2) | Abbreviation (3) | Typical substrates (4) | Total # (5) | Gene (02jul03) (6) | Length (#aas) (7) | # TMSs (8) | Nearest homologue in TCDB (9) | Evidence (10) |
|---|---|---|---|---|---|---|---|---|---|
| | total 30 | - ORF4 (8) | Hydrophobe/amphiphile substrates | 1 | Contig364gene3146 | 786 | 11 | 2.A.6.8.1(1) | 2 |
| 2.A.7 | Drug/metabolite transporter | DMT | Multiple drugs and dyes (mostly cationic) | | | | | | |
| | | - SMR (1) | Drugs | | Contig338gene297 | 109 | 4 | 2.A.7.1.3(1) | 3 |
| | | | | 2 | Contig374gene7258 | 123 | 5 | 2.A.7.1.3(1) | 3 |
| | | - BAT (2) | Unknown | | Contig356gene1583 | 362 | 11 | 2.A.7.2.1(1) | 3 |
| | | | | 2 | Contig375gene9035 | 143 | 5 | 2.A.7.2.1(1) | 2 |
| | | - DME (3) | Drugs, metabolites | | Contig375gene9463 | 297 | 10 | 2.A.7.3.2(1) | 2 |
| | | | | | Contig364gene3008 | 306 | 10 | 2.A.7.3.2(1) | 3 |
| | | | | | Contig374gene7235 | 312 | 10 | 2.A.7.3.2(1) | 3 |
| | | | | | Contig352gene1130 | 301 | 10 | 2.A.7.3.2(1) | 3 |
| | | | | | Contig375gene7949 | 297 | 10 | 2.A.7.3.2(1) | 3 |
| | | | | | Contig363gene2886 | 347 | 11 | 2.A.7.3.2(1) | 3 |
| | | | | | Contig375gene8660 | 337 | 11 | 2.A.7.3.3(1) | 3 |
| | | | | | Contig370gene4864 | 532 | 10 | 2.A.7.3.3(1) | 3 |
| | | | | | Contig361gene2346 | 300 | 10 | 2.A.7.3.4(1) | 3 |
| | | | | | Contig375gene8332 | 345 | 10 | 2.A.7.3.4(1) | 3 |
| | | | | | Contig348gene822 | 319 | 10 | 2.A.7.3.6(1) | 3 |
| | | | | 12 | Contig375gene7919 | 288 | 10 | 2.A.7.3.6(1) | 2 |
| | | - RarD (7) | Chloramphenicol | | Contig375gene7722 | 342 | 10 | 2.A.7.7.1(1) | 2 |
| | total 18 | | | 2 | Contig375gene8931 | 311 | 10 | 2.A.7.17.1(1) | 2 |
| 2.A.9 | Cytochrome oxidase bio-genesis | Oxa1 | Proteins | 1 | Contig375gene9312 | 555 | 4 | 2.A.9.3.1(1) | 2 |
| 2.A.10 | 2-Keto-3-deoxygluconate transporter | KDGT | 2-Keto-3-deoxygluconate | 1 | Contig356gene1558 | 327 | 10 | 2.A.10.1.1(1) | 2 |
| 2.A.11 | Citrate-$Mg^{2+}$ : $H^+$ (CitM) Citrate-$Ca^{2+}$ : $H^+$ (CitH) Symporter | CitMHS | Citrate | 1 | Contig375gene7824 | 485 | 11 | 2.A.11.1.1(1) | 3 |
| 2.A.12 | ATP : ADP antiporter | AAA | ATP, ADP | 1 | Contig375gene7893 | 453 | 10 | 2.A.12.3.1(1) | 3 |
| 2.A.14 | Lactate permease | LctP | Lactate | 1 | Contig372gene5556 | 566 | 16 | 2.A.14.1.2(1) | 2 |
| 2.A.19 | $Ca^{2+}$:cation antiporter | CaCA | $Ca^{2+}$ | 1 | Contig375gene7970 | 360 | 11 | 2.A.19.1.1(1) | 2 |
| 2.A.20 | Inorganic phosphate transporter | PiT | Inorganic phosphate | 1 | Contig344gene557 | 336 | 9 | 2.A.20.2.4(1) | 2 |
| 2.A.21 | Solute : sodium symporter | SSS | Sugars, amino acids, vitamins, nucleosides, inositols, iodide, urea | | Contig375gene7737 | 461 | 13 | 2.A.21.4.1(1) | 2 |
| | | | | | Contig375gene8758 | 683 | 14 | 2.A.21.7.1(1) | 2 |
| | | | | | Contig372gene5917 | 553 | 14 | 2.A.21.7.1(1) | 2 |
| | | | | | Contig359gene1964 | 478 | 13 | 2.A.21.8.1(1) | 2 |
| | | | | 5 | Contig374gene7322 | 967 | 4 | 2.A.21.9.1(1) | 3 |
| 2.A.23 | Dicarboxylate/amino acid : cation ($Na^+$ or $H^+$) symporter | DAACS | C4-dicarboxylates, acidic and neutral amino acids | 5 | Contig365gene3380 | 435 | 9 | 2.A.23.1.2(1) | 2 |
| | | | | | Contig369gene4393 | 430 | 8 | 2.A.23.1.2(1) | 2 |

Copyright © 2005 John Wiley & Sons, Ltd.

| | | | | | | | | | |
|---|---|---|---|---|---|---|---|---|---|
| | | | | | Contig375gene7654 | 467 | 10 | 2.A.23.1.3(1) | 2 |
| | | | | | Contig369gene4353 | 452 | 8 | 2.A.23.1.3(1) | 2 |
| | | | | | Contig374gene7480 | | | | |
| 2.A.24 | Citrate : cation symporter | CCS | Mono-, di-, and tricarboxylates | 1 | Contig373gene6794 | 448 | 13 | 2.A.24.2.5(1) | 2 |
| 2.A.36 | Monovalent cation : proton antiporter-1 | CPA1 | $Na^+/H^+$, $Na^+$ or $K^+/H^+$ | 1 | Contig373gene6805 | 435 | 12 | 2.A.36.6.1(1) | 3 |
| 2.A.37 | Monovalent cation : proton antiporter-2 | CPA2 | $Na^+/H^+$ or $K^+/H^+$ | | Contig358gene1749 | 404 | 12 | 2.A.37.1.1(2) | 2 |
| | | | | | Contig375gene9498 | 219 | 0 | 2.A.37.1.1(2) | 2 |
| | | | | | Contig375gene9499 | 604 | 13 | 2.A.37.1.1(2) | 2 |
| | | | | | Contig374gene7542 | 674 | 13 | 2.A.37.1.2(2) | 2 |
| | | | | | Contig375gene8748 | 408 | 13 | 2.A.37.1.2(2) | 2 |
| | | | | 6 | Contig375gene9414 | 406 | 12 | 2.A.37.1.2(2) | 3 |
| 2.A.40 | Nucleobase : cation symporter-2 | NCS2 | Nucleobases, urate | | Contig370gene4820 | 482 | 13 | 2.A.40.1.1(1) | 2 |
| | | | | | Contig372gene5621 | 453 | 13 | 2.A.40.1.1(1) | 3 |
| | | | | 3 | Contig371gene5375 | 445 | 14 | 2.A.40.3.1(1) | 2 |
| 2.A.45 | Arsenite–antimonite | ArsB | Arsenite, antimonite | 1 | Contig375gene8078 | 419 | 12 | 2.A.45.1.1(1) | 3 |
| 2.A.46 | Benzoate : $H^+$ symporter | BenE | Benzoate | 1 | Contig338gene295 | 395 | 12 | 2.A.46.1.1(1) | 2 |
| 2.A.47 | Divalent anion : $Na^+$ symporter | DASS | Dicarboxylates, phosphate, sulphate | | Contig369gene4367 | 181 | 5 | 2.A.47.3.1(1) | 2 |
| | | | | | Contig369gene4366 | 532 | 7 | 2.A.47.3.1(1) | 2 |
| | | | | | Contig365gene3403 | 507 | 15 | 2.A.47.3.3(1) | 2 |
| | | | | 4 | Contig373gene6264 | 530 | 15 | 2.A.47.3.3(1) | 3 |
| 2.A.49 | Ammonium transporter | Amt | Ammonium | | Contig375gene7868 | 400 | 0 | 2.A.49.X | 2 |
| | | | | 2 | Contig374gene7490 | 510 | 13 | 2.A.49.1.1(1) | 2 |
| 2.A.51 | Chromate ion transporter | CHR | Chromate, sulphate (uptake or efflux) | | Contig355gene1410 | 193 | 5 | 2.A.51.1.1(1) | 3 |
| | | | | | Contig371gene5134 | 390 | 12 | 2.A.51.1.1(1) | 1 |
| | | | | | Contig368gene4196 | 401 | 12 | 2.A.51.1.(0) | 1 |
| | | | | 4 | Contig375gene7933 | 408 | 11 | 2.A.51.1.2(1) | 3 |
| 2.A.52 | $Ni^{2+}$-$Co^{2+}$ transporter | NiCoT | $Ni^{2+}$, $Co^{2+}$ | 1 | Contig334gene209 | 278 | 7 | 2.A.52.1.2(1) | 3 |
| 2.A.53 | Sulphate permease | SulP | Sulphate | | Contig325gene78 | 603 | 11 | 2.A.53.1.4(1) | 2 |
| | | | | | Contig375gene8514 | 492 | 13 | 2.A.53.3.1(1) | 2 |
| | | | | | Contig365gene3367 | 599 | 11 | 2.A.53.4.1(1) | 2 |
| | | | | | Contig375gene8575 | 578 | 11 | 2.A.53.4.1(1) | 2 |
| | | | | 5 | Contig371gene5371 | 586 | 13 | 2.A.53.4.1(1) | 2 |
| 2.A.56 | Tripartite ATP-independent periplasmic transporter | TRAP-T | C4-dicarboxylates, acidic amino acids, sugars? | | Contig369gene4416 | 434 | 13 | 2.A.56.1.1(3) | 3 |
| | | | | | Contig361gene2330 | 327 | 1 | 2.A.56.1.1(3) | 3 |
| | | | | | Contig361gene2332 | 436 | 11 | 2.A.56.1.1(0) | 2 |
| | | | | | Contig369gene4417 | 343 | 1 | 2.A.56.1.1(3) | 2 |
| | | | | | Contig366gene3497 | 180 | 4 | 2.A.56.1.2(0) | 2 |
| | | | | 6 | Contig366gene3498 | 574 | 13 | 2.A.56.1.2(0) | 2 |

Copyright © 2005 John Wiley & Sons, Ltd.

**Table 3.** Continued

| Family (1) | Family (2) | Abbreviation (3) | Typical substrates (4) | Total # (5) | Gene (02jul03) (6) | Length (#aas) (7) | # TMSs (8) | Nearest homologue in TCDB (9) | Evidence (10) |
|---|---|---|---|---|---|---|---|---|---|
| 2.A.58 | Phosphate : $Na^+$ symporter | PNaS | Inorganic phosphate | | Contig353gene1224 | 632 | 9 | 2.A.58.2.1(1) | 2 |
| | | | | 2 | Contig374gene7545 | 558 | 9 | 2.A.58.2.1(1) | 2 |
| 2.A.59 | Arsenical resistance-3 | ACR3 | Arsenite | 1 | Contig359gene2078 | 354 | 10 | 2.A.59.1.1(1) | 3 |
| 2.A.64 | Twin arginine targeting | Tat | Redox proteins | | Contig367gene3817 | 77 | 1 | 2.A.64.1.1(4) | 3 |
| | | | | | Contig367gene3818 | 168 | 1 | 2.A.64.1.1(4) | 3 |
| | | | | | Contig367gene3819 | 260 | 5 | 2.A.64.1.1(4) | 2 |
| | | | | 4 | Contig367gene3820 | 401 | 1 | 2.A.64.1.1(4) | 2 |
| 2.A.66 | Multidrug/oligosaccharidyl-lipid/polysaccharide | MOP | Drugs, lipid-linked oligosaccharide precursors | | | | | | |
| | | - MATE (1) | Drugs | | Contig362gene2523 | 449 | 12 | 2.A.66.1.1(1) | 2 |
| | | | | | Contig367gene3916 | 455 | 12 | 2.A.66.1.1(1) | 3 |
| | | | | 3 | Contig375gene8978 | 492 | 12 | 2.A.66.1.3(1) | 3 |
| | | - PST (2) | Polysaccharides | 1 | Contig366gene3637 | 419 | 12 | 2.A.66.2.4(1) | 2 |
| | | - MVF (4) | Unknown | 1 | Contig372gene5715 | 534 | 14 | 2.A.66.4.1(1) | 2 |
| 2.A.67 | Oligopeptide transporter | OPT | Peptides | | Contig372gene5640 | 668 | 17 | 2.A.67.3.1(1) | 3 |
| | | | | 2 | Contig363gene2777 | 676 | 18 | 2.A.67.4.1(1) | 2 |
| 2.A.69 | Auxin efflux carrier | AEC | Auxin (efflux) | | Contig356gene1506 | 293 | 10 | 2.A.69.1.1(1) | 3 |
| | | | | 2 | Contig375gene9534 | 351 | 10 | 2.A.69.2.1(1) | 3 |
| 2.A.72 | $K^+$ uptake permease | KUP | $K^+$ (uptake) | 1 | Contig349gene850 | 656 | 11 | 2.A.72.1.1(1) | 2 |
| 2.A.75 | L-Lysine exporter | LysE | Basic amino acids | 1 | Contig371gene5136 | 216 | 6 | 2.A.75.1.1(1) | 2 |
| 2.A.76 | Resistance to homoserine/threonine | RhtB | Neutral amino acids and their derivatives | | Contig355gene1462 | 205 | 5 | 2.A.76.1.1(1) | 3 |
| | | | | | Contig362gene2710 | 223 | 6 | 2.A.76.1.1(1) | 3 |
| | | | | | Contig355gene1382 | 209 | 6 | 2.A.76.1.1(1) | 3 |
| | | | | | Contig363gene2791 | 212 | 6 | 2.A.76.1.1(1) | 3 |
| | | | | | Contig371gene5511 | 208 | 6 | 2.A.76.1.1(1) | 3 |
| | | | | | Contig374gene7486 | 214 | 6 | 2.A.76.1.1(1) | 2 |
| | | | | | Contig375gene7623 | 212 | 6 | 2.A.76.1.1(1) | 3 |
| | | | | | Contig372gene5890 | 212 | 6 | 2.A.76.1.1(1) | 3 |
| | | | | | Contig373gene6271 | 205 | 5 | 2.A.76.1.2(1) | 3 |
| | | | | | Contig373gene6137 | 203 | 6 | 2.A.76.1.2(1) | 3 |
| | | | | 11 | Contig372gene5541 | 204 | 6 | 2.A.76.1.2(1) | 3 |
| 2.A.78 | Branched chain amino acid exporter | LIV-E | Carboxylates, amino acids, amines (efflux) | 1 | Contig352gene1134 | 265 | 4 | 2.A.78.1.1(2) | 3 |
| 2.A.80 | Tricarboxylate transporter | TTT | Tricarboxylate | | Contig373gene6710 | 326 | 1 | 2.A.80.1.1(3) | 3 |
| | | | | | Contig364gene3003 | 322 | 1 | 2.A.80.1.1(3) | 3 |
| | | | | | Contig364gene2995 | 320 | 1 | 2.A.80.1.1(3) | 3 |
| | | | | | Contig358gene1831 | 337 | 0 | 2.A.80.1.1(3) | 3 |
| | | | | | Contig370gene4730 | 554 | 1 | 2.A.80.1.1(3) | 3 |

Copyright © 2005 John Wiley & Sons, Ltd.

| | | | | |
|---|---|---|---|---|
| Contig345gene580 | 327 | 4 | 2.A.80.1.1(3) | 3 |
| Contig360gene2191 | 326 | 1 | 2.A.80.1.1(3) | 3 |
| Contig373gene6749 | 322 | 3 | 2.A.80.1.1(3) | 3 |
| Contig373gene6096 | 328 | 3 | 2.A.80.1.1(3) | 3 |
| Contig373gene6578 | 330 | 0 | 2.A.80.1.1(3) | 3 |
| Contig371gene5514 | 328 | 4 | 2.A.80.1.1(3) | 3 |
| Contig371gene5517 | 325 | 2 | 2.A.80.1.1(3) | 3 |
| Contig373gene6354 | 322 | 0 | 2.A.80.1.1(3) | 3 |
| Contig370gene4900 | 323 | 4 | 2.A.80.1.1(3) | 3 |
| Contig357gene1683 | 334 | 4 | 2.A.80.1.1(3) | 3 |
| Contig375gene9115 | 328 | 2 | 2.A.80.1.1(3) | 3 |
| Contig358gene1825 | 327 | 1 | 2.A.80.1.1(3) | 3 |
| Contig373gene6531 | 321 | 0 | 2.A.80.1.1(3) | 3 |
| Contig357gene1608 | 332 | 1 | 2.A.80.1.1(3) | 3 |
| Contig372gene5872 | 334 | 0 | 2.A.80.1.1(3) | 3 |
| Contig335gene234 | 311 | 0 | 2.A.80.1.1(3) | 2 |
| Contig361gene2500 | 327 | 1 | 2.A.80.1.1(3) | 3 |
| Contig341gene384 | 336 | 1 | 2.A.80.1.1(3) | 3 |
| Contig353gene1176 | 332 | 4 | 2.A.80.1.1(3) | 3 |
| Contig374gene7144 | 366 | 0 | 2.A.80.1.1(3) | 3 |
| Contig374gene7146 | 327 | 0 | 2.A.80.1.1(3) | 3 |
| Contig373gene6763 | 348 | 0 | 2.A.80.1.1(3) | 3 |
| Contig345gene622 | 323 | 1 | 2.A.80.1.1(3) | 3 |
| Contig345gene628 | 363 | 1 | 2.A.80.1.1(3) | 3 |
| Contig357gene1594 | 329 | 2 | 2.A.80.1.1(3) | 3 |
| Contig366gene3580 | 336 | 3 | 2.A.80.1.1(3) | 3 |
| Contig354gene1306 | 353 | 2 | 2.A.80.1.1(3) | 3 |
| Contig371gene5098 | 332 | 2 | 2.A.80.1.1(3) | 3 |
| Contig371gene5502 | 341 | 0 | 2.A.80.1.1(3) | 3 |
| Contig370gene4704 | 500 | 0 | 2.A.80.1.1(3) | 2 |
| Contig370gene4705 | 325 | 0 | 2.A.80.1.1(3) | 3 |
| Contig375gene8996 | 328 | 1 | 2.A.80.1.1(3) | 3 |
| Contig358gene1912 | 333 | 0 | 2.A.80.1.1(3) | 3 |
| Contig375gene7952 | 334 | 0 | 2.A.80.1.1(3) | 3 |
| Contig370gene4597 | 333 | 0 | 2.A.80.1.1(3) | 3 |
| Contig375gene8980 | 500 | 12 | 2.A.80.1.1(3) | 2 |
| Contig375gene8884 | 330 | 2 | 2.A.80.1.1(3) | 3 |
| Contig356gene1500 | 337 | 2 | 2.A.80.1.1(3) | 3 |
| Contig373gene6518 | 331 | 0 | 2.A.80.1.1(3) | 3 |
| Contig354gene1258 | 345 | 2 | 2.A.80.1.1(3) | 3 |
| Contig375gene8567 | 348 | 0 | 2.A.80.1.1(3) | 3 |
| Contig375gene8579 | 353 | 0 | 2.A.80.1.1(3) | 3 |
| Contig371gene5324 | 346 | 4 | 2.A.80.1.1(3) | 3 |

Copyright © 2005 John Wiley & Sons, Ltd.

**Table 3.** Continued

| Family (1) | Family (2) | Abbreviation (3) | Typical substrates (4) | Total # (5) | Gene (02jul03) (6) | Length (#aas) (7) | # TMSs (8) | Nearest homologue in TCDB (9) | Evidence (10) |
|---|---|---|---|---|---|---|---|---|---|
| | | | | | Contig367gene3835 | 323 | 1 | 2.A.80.1.1(3) | 3 |
| | | | | | Contig364gene3052 | 320 | 0 | 2.A.80.1.1(3) | 3 |
| | | | | | Contig364gene3101 | 382 | 2 | 2.A.80.1.1(3) | 3 |
| | | | | | Contig373gene6242 | 333 | 0 | 2.A.80.1.1(3) | 3 |
| | | | | | Contig373gene6267 | 322 | 1 | 2.A.80.1.1(3) | 3 |
| | | | | | Contig373gene6280 | 330 | 1 | 2.A.80.1.1(3) | 3 |
| | | | | | Contig373gene6586 | 331 | 4 | 2.A.80.1.1(3) | 3 |
| | | | | | Contig375gene7775 | 323 | 1 | 2.A.80.1.1(3) | 3 |
| | | | | | Contig371gene5154 | 322 | 1 | 2.A.80.1.1(3) | 3 |
| | | | | | Contig371gene5178 | 326 | 2 | 2.A.80.1.1(3) | 3 |
| | | | | | Contig371gene5421 | 318 | 3 | 2.A.80.1.1(3) | 3 |
| | | | | | Contig371gene5426 | 326 | 1 | 2.A.80.1.1(3) | 3 |
| | | | | | Contig371gene5429 | 320 | 1 | 2.A.80.1.1(3) | 3 |
| | | | | | Contig371gene5443 | 325 | 2 | 2.A.80.1.1(3) | 3 |
| | | | | | Contig373gene6160 | 341 | 1 | 2.A.80.1.1(3) | 3 |
| | | | | | Contig373gene6671 | 329 | 1 | 2.A.80.1.1(3) | 3 |
| | | | | | Contig373gene6675 | 320 | 0 | 2.A.80.1.1(3) | 3 |
| | | | | | Contig372gene6001 | 328 | 1 | 2.A.80.1.1(3) | 3 |
| | | | | | Contig375gene9477 | 330 | 3 | 2.A.80.1.1(3) | 3 |
| | | | | | Contig375gene9392 | 385 | 1 | 2.A.80.1.1(3) | 3 |
| | | | | | Contig375gene7729 | 322 | 1 | 2.A.80.1.1(3) | 2 |
| | | | | | Contig375gene7731 | 504 | 13 | 2.A.80.1.1(3) | 2 |
| | | | | | Contig375gene8159 | 333 | 1 | 2.A.80.1.1(3) | 2 |
| | | | | | Contig375gene8161 | 551 | 12 | 2.A.80.1.1(3) | 2 |
| | | | | | Contig375gene8171 | 329 | 0 | 2.A.80.1.1(3) | 3 |
| | | | | 74 | Contig375gene8944 | 513 | 11 | 2.A.80.1.1(3) | 2 |
| 2.A.81 | Aspartate : alanine exchanger | AAE | Aspartate, alanine | | Contig344gene526 | 561 | 11 | 2.A.81.1.1(1) | 2 |
| | | | | 2 | Contig375gene7947 | 567 | 11 | 2.A.81.1.1(1) | 2 |
| 2.C. Ion-gradient-driven energizers | | | | | | | | | |
| 2.C.1 | TonB-ExbB-ExbD/TolA-TolQ-TolR family of auxiliary proteins for energization of outer membrane receptor (OMR)-mediated active transport | TonB | $H^+$?, drives solute uptake across outer bacterial membranes | | Contig375gene8338 | 243 | 3 | 2.C.1.1.1(3) | 3 |
| | | | | | Contig366gene3670 | 227 | 3 | 2.C.1.2.1(6) | 2 |
| | | | | | Contig366gene3671 | 145 | 1 | 2.C.1.2.1(6) | 3 |
| | | | | 5 | Contig366gene3673 | 446 | 1 | 2.C.1.2.1(6) | 3 |

Copyright © 2005 John Wiley & Sons, Ltd.

| | | | | | | | | | |
|---|---|---|---|---|---|---|---|---|---|
| 3.A P-P bond hydrolysis-driven transporters | | | | | | | | | |
| 3.A.1 | ATP-binding cassette | ABC | All sorts of inorganic and organic molecules of small, intermediate, and large sizes, from simple ions to macromolecules | | | | | | |
| | | | | | Contig375gene8339 | 137 | 1 | 2.C.1.1.1(3) | 3 |
| | | - CUT1 (1) | Sugars, metabolites | | | | | | |
| | | | | | Contig360gene2245 | 293 | 6 | 3.A.1.1.3(4) | 2 |
| | | | | | Contig360gene2246 | 282 | 6 | 3.A.1.1.3(4) | 2 |
| | | | | | Contig360gene2247 | 367 | 1 | 3.A.1.1.3(4) | 2 |
| | | | | | Contig362gene2677 | 395 | 1 | 3.A.1.1.X | 2 |
| | | | | | Contig362gene2678 | 366 | 0 | 3.A.1.1.X | 2 |
| | | | | | Contig362gene2679 | 294 | 6 | 3.A.1.1.X | 2 |
| | | | | | Contig362gene2680 | 276 | 6 | 3.A.1.1.X | 2 |
| | | | | | Contig362gene2682 | 580 | 1 | 3.A.1.1.X | 3 |
| | | | | | Contig375gene7943 | 352 | 1 | 3.A.1.1.12(4) | 2 |
| | | | | | Contig365gene3399 | 279 | 0 | 3.A.1.1.16(4) | 2 |
| | | | | | Contig375gene9297 | 464 | 0 | 3.A.1.1.X | 3 |
| | | | | | Contig375gene9298 | 371 | 1 | 3.A.1.1.X | 2 |
| | | | | | Contig375gene9299 | 310 | 6 | 3.A.1.1.X | 2 |
| | | | | 15 | Contig375gene9300 | 295 | 6 | 3.A.1.1.X | 2 |
| | | - CUT2 (2) | Sugars, metabolites | | Contig349gene903 | 298 | 9 | 3.A.1.2.1(4) | 3 |
| | | | | | Contig370gene4724 | 537 | 0 | 3.A.1.2.X | 2 |
| | | | | | Contig370gene4725 | 364 | 10 | 3.A.1.2.X | 3 |
| | | | | 4 | Contig370gene4726 | 306 | 9 | 3.A.1.X | 3 |
| | | - PAAT (3) | Polar amino acids | | Contig346gene661 | 302 | 1 | 3.A.1.3.4(4) | 2 |
| | | | | | Contig369gene4394 | 303 | 0 | 3.A.1.3.4(4) | 3 |
| | | | | | Contig359gene1941 | 302 | 1 | 3.A.1.3.4(4) | 2 |
| | | | | | Contig346gene653 | 282 | 1 | 3.A.1.3.4(4) | 2 |
| | | | | | Contig346gene654 | 231 | 5 | 3.A.1.3.4(4) | 2 |
| | | | | | Contig346gene655 | 447 | 6 | 3.A.1.3.4(4) | 2 |
| | | | | | Contig346gene656 | 249 | 0 | 3.A.1.3.4(4) | 2 |
| | | | | | Contig374gene7255 | 304 | 0 | 3.A.1.3.4(4) | 2 |
| | | | | | Contig338gene294 | 310 | 1 | 3.A.1.3.4(4) | 2 |
| | | | | | Contig359gene1987 | 299 | 0 | 3.A.1.3.4(4) | 2 |
| | | | | | Contig359gene1988 | 242 | 5 | 3.A.1.3.4(4) | 2 |
| | | | | | Contig359gene1989 | 227 | 5 | 3.A.1.3.4(4) | 2 |
| | | | | | Contig359gene1990 | 244 | 0 | 3.A.1.3.4(4) | 2 |
| | | | | 14 | Contig371gene5478 | 274 | 0 | 3.A.1.3.10(3) | 2 |
| | | - HAAT (4) | Hydrophobic amino acids | | Contig350gene948 | 384 | 9 | 3.A.1.4.1(6) | 2 |
| | | | | | Contig350gene949 | 258 | 0 | 3.A.1.4.1(6) | 2 |
| | | | | | Contig350gene950 | 238 | 0 | 3.A.1.4.1(6) | 2 |
| | | | | | Contig374gene7171 | 361 | 0 | 3.A.1.4.X | 3 |
| | | | | | Contig374gene7172 | 285 | 7 | 3.A.1.4.X | 2 |

Copyright © 2005 John Wiley & Sons, Ltd.

**Table 3.** Continued

| Family (1) | Family (2) | Abbreviation (3) | Typical substrates (4) | Total # (5) | Gene (02jul03) (6) | Length (#aas) (7) | # TMSs (8) | Nearest homologue in TCDB (9) | Evidence (10) |
|---|---|---|---|---|---|---|---|---|---|
| | | | | | Contig374gene7174 | 255 | 0 | 3.A.1.4.1(6) | 2 |
| | | | | | Contig361gene2483 | 437 | 11 | 3.A.1.4.1(6) | 3 |
| | | | | | Contig361gene2484 | 259 | 0 | 3.A.1.4.1(6) | 2 |
| | | | | | Contig361gene2485 | 237 | 0 | 3.A.1.4.1(6) | 2 |
| | | | | | Contig355gene1439 | 479 | 1 | 3.A.1.4.1(6) | 2 |
| | | | | | Contig355gene1440 | 308 | 9 | 3.A.1.4.1(6) | 2 |
| | | | | | Contig370gene4963 | 425 | 1 | 3.A.1.4.1(6) | 3 |
| | | | | | Contig355gene1441 | 424 | 11 | 3.A.1.4.1(6) | 2 |
| | | | | | Contig355gene1442 | 255 | 0 | 3.A.1.4.1(6) | 2 |
| | | | | | Contig355gene1443 | 233 | 0 | 3.A.1.4.1(6) | 2 |
| | | | | | Contig340gene370 | 398 | 3 | 3.A.1.4.1(6) | 3 |
| | | | | | Contig375gene8086 | 287 | 7 | 3.A.1.4.X | 2 |
| | | | | | Contig375gene8087 | 342 | 10 | 3.A.1.4.X | 2 |
| | | | | | Contig375gene8088 | 254 | 1 | 3.A.1.4.1(6) | 2 |
| | | | | | Contig375gene8089 | 235 | 2 | 3.A.1.4.1(6) | 2 |
| | | | | | Contig375gene8090 | 390 | 1 | 3.A.1.4.X | 3 |
| | | | | | Contig349gene849 | 238 | 0 | 3.A.1.4.1(6) | 2 |
| | | | | | Contig372gene5996 | 379 | 2 | 3.A.1.4.1(6) | 3 |
| | | | | | Contig374gene7472 | 313 | 0 | 3.A.1.4.1(6) | 2 |
| | | | | | Contig374gene7473 | 304 | 8 | 3.A.1.4.1(6) | 2 |
| | | | | | Contig374gene7474 | 358 | 10 | 3.A.1.4.X | 3 |
| | | | | | Contig374gene7476 | 271 | 0 | 3.A.1.4.1(6) | 2 |
| | | | | | Contig375gene9380 | 257 | 0 | 3.A.1.4.X | 2 |
| | | | | | Contig375gene9381 | 241 | 0 | 3.A.1.4.1(6) | 2 |
| | | | | | Contig375gene9382 | 402 | 1 | 3.A.1.4.X | 3 |
| | | | | | Contig375gene9387 | 382 | 1 | 3.A.1.4.X | 3 |
| | | | | | Contig375gene9388 | 350 | 9 | 3.A.1.4.1(6) | 3 |
| | | | | | Contig375gene9389 | 617 | 10 | 3.A.1.4.1(6) | 2 |
| | | | | | Contig375gene9390 | 247 | 0 | 3.A.1.4.X | 2 |
| | | | | | Contig361gene2481 | 416 | 1 | 3.A.1.4.X | 3 |
| | | | | | Contig361gene2482 | 323 | 8 | 3.A.1.4.2(5) | 3 |
| | | | | | Contig375gene9184 | 288 | 7 | 3.A.1.4.2(5) | 3 |
| | | | | | Contig366gene3541 | 389 | 1 | 3.A.1.4.2(5) | 3 |
| | | | | | Contig374gene6823 | 263 | 0 | 3.A.1.4.2(5) | 2 |
| | | | | | Contig350gene946 | 401 | 1 | 3.A.1.4.3(4) | 2 |
| | | | | | Contig375gene9383 | 294 | 8 | 3.A.1.4.3(4) | 2 |
| | | | | | Contig375gene9384 | 344 | 9 | 3.A.1.4.3(4) | 3 |
| | | | | | Contig375gene9412 | 383 | 1 | 3.A.1.4.3(4) | 2 |
| | | | | | Contig358gene1906 | 412 | 1 | 3.A.1.4.4(5) | 3 |
| | | | | 45 | Contig370gene5000 | 230 | 0 | 3.A.1.4.4(5) | 3 |
| | | - PepT (5) | Peptide, opine, nickel | | Contig373gene6123 | 348 | 0 | 3.A.1.5.X | 2 |

Copyright © 2005 John Wiley & Sons, Ltd. 

| | | | | | | | |
|---|---|---|---|---|---|---|---|
| | | | Contig373gene6124 | 337 | 0 | 3.A.1.5.X | 2 |
| | | | Contig373gene6120 | 527 | 0 | 3.A.1.5.X | 3 |
| | | | Contig373gene6122 | 299 | 5 | 3.A.1.5.X | 2 |
| | | | Contig336gene262 | 306 | 0 | 3.A.1.5.2(5) | 2 |
| | | | Contig336gene263 | 300 | 6 | 3.A.1.5.X | 2 |
| | | | Contig336gene265 | 275 | 0 | 3.A.1.5.2(5) | 2 |
| | | | Contig362gene2744 | 661 | 1 | 3.A.1.5.2(5) | 3 |
| | | | Contig362gene2745 | 349 | 6 | 3.A.1.5.2(5) | 2 |
| | | | Contig362gene2746 | 376 | 6 | 3.A.1.5.2(5) | 2 |
| | | | Contig362gene2747 | 549 | 0 | 3.A.1.5.2(5) | 2 |
| | | | Contig370gene4823 | 344 | 0 | 3.A.1.5.2(5) | 3 |
| | | | Contig374gene7247 | 318 | 6 | 3.A.1.5.X | 2 |
| | | | Contig374gene7248 | 308 | 6 | 3.A.1.5.X | 2 |
| | | | Contig374gene7249 | 575 | 1 | 3.A.1.5.X | 2 |
| | | | Contig374gene7250 | 325 | 0 | 3.A.1.5.X | 2 |
| | | | Contig374gene7251 | 367 | 0 | 3.A.1.5.X | 2 |
| | | | Contig355gene1375 | 545 | 1 | 3.A.1.5.4(5) | 2 |
| | | | Contig357gene1662 | 259 | 1 | 3.A.1.5.3(5) | 2 |
| | | | Contig340gene353 | 535 | 1 | 3.A.1.5.X | 2 |
| | | | Contig340gene354 | 325 | 6 | 3.A.1.5.X | 2 |
| | | | Contig340gene355 | 309 | 5 | 3.A.1.5.X | 2 |
| | | | Contig340gene356 | 332 | 0 | 3.A.1.5.X | 2 |
| | | | Contig340gene357 | 354 | 0 | 3.A.1.5.X | 2 |
| | | | Contig358gene1735 | 289 | 1 | 3.A.1.5.X | 3 |
| | | | Contig358gene1736 | 347 | 6 | 3.A.1.5.X | 2 |
| | | | Contig358gene1737 | 279 | 6 | 3.A.1.5.X | 2 |
| | | | Contig358gene1738 | 547 | 0 | 3.A.1.5.X | 2 |
| | | | Contig361gene2374 | 586 | 0 | 3.A.1.5.X | 2 |
| | | | Contig361gene2375 | 316 | 6 | 3.A.1.5.X | 2 |
| | | | Contig361gene2376 | 295 | 5 | 3.A.1.5.X | 2 |
| | | | Contig361gene2377 | 359 | 0 | 3.A.1.5.X | 2 |
| | | 33 | Contig361gene2378 | 337 | 0 | 3.A.1.5.X | 2 |
| - SulT (6) | Sulphate, tungstate | | Contig351gene1029 | 335 | 1 | 3.A.1.6.1(5) | 2 |
| | | | Contig351gene1036 | 335 | 6 | 3.A.1.6.1(5) | 2 |
| | | | Contig351gene1037 | 305 | 6 | 3.A.1.6.1(5) | 2 |
| | | | Contig351gene1038 | 367 | 0 | 3.A.1.6.1(5) | 2 |
| | | | Contig374gene6978 | 279 | 0 | 3.A.1.6.1(5) | 2 |
| | | 6 | Contig367gene3795 | 232 | 0 | 3.A.1.6.3(4) | 2 |
| - PhoT (7) | Phosphate | | Contig362gene2718 | 343 | 1 | 3.A.1.7.1(4) | 2 |
| | | | Contig362gene2719 | 321 | 6 | 3.A.1.7.1(4) | 2 |
| | | | Contig362gene2720 | 300 | 6 | 3.A.1.7.1(4) | 2 |
| | | | Contig362gene2721 | 262 | 0 | 3.A.1.7.1(4) | 2 |
| | | | Contig372gene5607 | 333 | 1 | 3.A.1.7.1(4) | 2 |
| | | 6 | Contig375gene8133 | 355 | 3 | 3.A.1.7.1(4) | 2 |

Copyright © 2005 John Wiley & Sons, Ltd.

**Table 3.** Continued

| Family (1) | Family (2) | Abbreviation (3) | Typical substrates (4) | Total # (5) | Gene (02jul03) (6) | Length (#aas) (7) | # TMSs (8) | Nearest homologue in TCDB (9) | Evidence (10) |
|---|---|---|---|---|---|---|---|---|---|
| | | - MolT (8) | Molybdate | | Contig372gene5726 | 232 | 5 | 3.A.1.8.1(3) | 3 |
| | | | | | Contig375gene7940 | 272 | 6 | 3.A.1.8.1(3) | 2 |
| | | | | | Contig365gene3396 | 258 | 1 | 3.A.1.8.1(3) | 2 |
| | | | | 4 | Contig365gene3398 | 238 | 5 | 3.A.1.8.1(3) | 2 |
| | | - PhnT (9) | Phosphonate | | Contig349gene904 | 264 | 0 | 3.A.1.9.1(3) | 2 |
| | | | | | Contig370gene4744 | 326 | 0 | 3.A.1.9.1(3) | 2 |
| | | | | | Contig370gene4745 | 349 | 6 | 3.A.1.9.1(3) | 2 |
| | | | | | Contig372gene5787 | 279 | 0 | 3.A.1.9.1(3) | 2 |
| | | | | | Contig372gene5788 | 292 | 1 | 3.A.1.9.1(3) | 3 |
| | | | | 6 | Contig372gene5789 | 266 | 5 | 3.A.1.9.1(3) | 2 |
| | | - POPT(11) | Polyamine, opine, phosphonate | | Contig364gene3011 | 364 | 0 | 3.A.1.11.1(4) | 2 |
| | | | | | Contig364gene3012 | 338 | 6 | 3.A.1.11.1(4) | 2 |
| | | | | | Contig364gene3013 | 260 | 6 | 3.A.1.11.1(4) | 2 |
| | | | | | Contig364gene3014 | 362 | 1 | 3.A.1.11.X | 2 |
| | | | | | Contig371gene5479 | 259 | 6 | 3.A.1.11.1(4) | 3 |
| | | | | | Contig375gene7939 | 340 | 1 | 3.A.1.11.X | 3 |
| | | | | | Contig375gene7942 | 291 | 6 | 3.A.1.11.X | 3 |
| | | | | 8 | Contig372gene5727 | 229 | 0 | 3.A.1.11.2(4) | 2 |
| | | - QAT (12) | Quaternary amine | | Contig370gene4824 | 217 | 5 | 3.A.1.12.6(3) | 3 |
| | | | | | Contig357gene1649 | 516 | 6 | 3.A.1.12.3(4) | 2 |
| | | | | | Contig370gene4872 | 316 | 1 | 3.A.1.12.4(4) | 2 |
| | | | | | Contig370gene4873 | 216 | 5 | 3.A.1.12.4(4) | 2 |
| | | | | 5 | Contig370gene4874 | 398 | 0 | 3.A.1.12.4(4) | 2 |
| | | - VB12T(13) | Vitamin $B_{12}$ | 1 | Contig366gene3515 | 300 | 0 | 3.A.1.13.1(3) | 2 |
| | | - FeCT (14) | Iron chelate | | Contig366gene3586 | 335 | 9 | 3.A.1.14.X | 2 |
| | | | | | Contig366gene3587 | 269 | 0 | 3.A.1.14.X | 2 |
| | | | | | Contig373gene6359 | 283 | 1 | 3.A.1.14.5(3) | 2 |
| | | | | | Contig373gene6360 | 333 | 9 | 3.A.1.14.5(3) | 2 |
| | | | | 5 | Contig373gene6361 | 261 | 0 | 3.A.1.14.5(3) | 2 |
| | | - MZT (15) | Manganese, zinc, iron chelate | 1 | Contig375gene9102 | 264 | 0 | 3.A.1.15.1(3) | 3 |
| | | - NitT (16) | Nitrate, nitrite, cyanate | | Contig374gene6840 | 434 | 3 | 3.A.1.16.X | 2 |
| | | | | | Contig374gene6841 | 303 | 6 | 3.A.1.16.1(4) | 2 |
| | | | | | Contig374gene6842 | 267 | 1 | 3.A.1.16.1(4) | 2 |
| | | | | | Contig360gene2192 | 347 | 2 | 3.A.1.16.2(3) | 3 |
| | | | | | Contig360gene2193 | 270 | 0 | 3.A.1.16.2(3) | 2 |
| | | | | | Contig362gene2587 | 341 | 1 | 3.A.1.16.2(3) | 3 |
| | | | | | Contig362gene2588 | 347 | 8 | 3.A.1.16.2(3) | 2 |
| | | | | | Contig362gene2589 | 262 | 0 | 3.A.1.16.X | 2 |
| | | | | | Contig350gene955 | 317 | 3 | 3.A.1.16.X | 3 |

Copyright © 2005 John Wiley & Sons, Ltd.

| | | | | | | | | |
|---|---|---|---|---|---|---|---|---|
| | | | | Contig350gene956 | 291 | 6 | 3.A.1.16.2(3) | 2 |
| | | | | Contig350gene957 | 259 | 0 | 3.A.1.16.2(3) | 2 |
| | | | | Contig364gene3098 | 256 | 1 | 3.A.1.16.2(3) | 2 |
| | | | | Contig375gene8294 | 304 | 0 | 3.A.1.16.2(3) | 2 |
| | | | 14 | Contig367gene3893 | 581 | 12 | 3.A.1.16.3(4) | 3 |
| | - TauT (17) | Taurine | | Contig360gene2194 | 263 | 5 | 3.A.1.17.1(3) | 2 |
| | | | | Contig351gene1030 | 345 | 1 | 3.A.1.17.2(3) | 2 |
| | | | | Contig351gene1032 | 268 | 6 | 3.A.1.17.2(3) | 2 |
| | | | | Contig351gene1033 | 291 | 0 | 3.A.1.17.2(3) | 2 |
| | | | | Contig350gene955 | 317 | 3 | 3.A.1.17.1(3) | 3 |
| | | | | Contig359gene1981 | 352 | 1 | 3.A.1.17.1(3) | 3 |
| | | | | Contig359gene1982 | 281 | 0 | 3.A.1.17.1(3) | 2 |
| | | | | Contig359gene1983 | 259 | 6 | 3.A.1.17.X | 2 |
| | | | | Contig367gene3894 | 448 | 0 | 3.A.1.17.1(3) | 2 |
| | | | | Contig364gene3097 | 348 | 1 | 3.A.1.17.X | 3 |
| | | | | Contig375gene8293 | 341 | 0 | 3.A.1.17.X | 3 |
| | | | | Contig364gene3099 | 298 | 7 | 3.A.1.17.X | 3 |
| | | | 13 | Contig375gene8295 | 388 | 7 | 3.A.1.17.X | 3 |
| uptake—total 181 | - BIT (20) | $Fe^{3+}$ | 1 | Contig375gene7762 | 371 | 1 | 3.A.1.20.1(6) | 3 |
| | - CPSE (101) | Capsular polysaccharides | | Contig375gene8669 | 262 | 6 | 3.A.1.101.1(2) | 2 |
| | | | 2 | Contig375gene8670 | 221 | 0 | 3.A.1.101.1(2) | 2 |
| | - LOSE (102) | Lipo-oligosaccharide | | Contig358gene1866 | 919 | 6 | 3.A.1.102.1(2) | 2 |
| | | | | Contig355gene1354 | 303 | 1 | 3.A.1.102.1(2) | 2 |
| | | | | Contig355gene1355 | 285 | 6 | 3.A.1.102.1(2) | 2 |
| | | | | Contig359gene2065 | 328 | 1 | 3.A.1.102.1(2) | 2 |
| | | | | Contig367gene3801 | 316 | 0 | 3.A.1.102.1(2) | 2 |
| | | | | Contig362gene2639 | 83 | 0 | 3.A.1.102.1(2) | 3 |
| | | | 7 | Contig362gene2647 | 372 | 7 | 3.A.1.102.1(2) | 3 |
| | - DrugE1 (105) | Drugs | | Contig359gene2066 | 384 | 6 | 3.A.1.105.2(2) | 3 |
| | | | 2 | Contig375gene9090 | 316 | 1 | 3.A.1.105.2(2) | 2 |
| | - HemeE (107) | Heme | | Contig375gene8794 | 207 | 0 | 3.A.1.107.1(3) | 2 |
| | | | | Contig375gene8795 | 228 | 6 | 3.A.1.107.1(3) | 2 |
| | | | | Contig375gene8796 | 245 | 6 | 3.A.1.107.1(3) | 2 |
| | | | | Contig357gene1721 | 211 | 0 | 3.A.1.107.1(3) | 2 |
| | | | | Contig357gene1722 | 222 | 6 | 3.A.1.107.1(3) | 3 |
| | | | 6 | Contig357gene1723 | 259 | 6 | 3.A.1.107.1(3) | 2 |
| | - Prot1E (109) | Proteins | 1 | Contig370gene4796 | 767 | 6 | 3.A.1.109.2(1) | 2 |
| | - Prot2E (110) | Proteins | 1 | Contig372gene5991 | 232 | 1 | 3.A.1.110.2(1) | 3 |
| | - Drug RA1 (120) | Drugs | | Contig342gene443 | 540 | 0 | 3.A.1.120.1(1) | 2 |
| | | | | Contig355gene1406 | 659 | 0 | 3.A.1.120.1(1) | 2 |
| | | | | Contig373gene6751 | 536 | 0 | 3.A.1.120.2(1) | 2 |

Copyright © 2005 John Wiley & Sons, Ltd.

**Table 3.** Continued

| Family (1) | Family (2) | Abbreviation (3) | Typical substrates (4) | Total # (5) | Gene (02jul03) (6) | Length (#aas) (7) | # TMSs (8) | Nearest homologue in TCDB (9) | Evidence (10) |
|---|---|---|---|---|---|---|---|---|---|
| | | | | | Contig353gene1211 | 554 | 0 | 3.A.1.120.3(1) | 3 |
| | | | | 5 | Contig367gene3831 | 670 | 0 | 3.A.1.120.4(1) | 2 |
| | | - Drug RA2 (121) | Drugs | 1 | Contig336gene249 | 347 | 0 | 3.A.1.121.2(1) | 2 |
| | | - MacB (122) | Macrolide | 1 | Contig353gene1239 | 234 | 0 | 3.A.1.122.1(1) | 2 |
| | | - LPT (125) | Lipoproteins | | Contig361gene2469 | 208 | 0 | 3.A.1.125.1(3) | 2 |
| | | | | | Contig370gene5060 | 416 | 5 | 3.A.1.125.1(3) | 2 |
| | | | | 3 | Contig370gene5061 | 249 | 0 | 3.A.1.125.1(3) | 2 |
| | export -total 30 | - HMT (210) | Heavy metals | | Contig355gene1389 | 610 | 6 | 3.A.1.210.2(1) | 2 |
| | total 211 | | | 2 | Contig359gene1980 | 630 | 7 | 3.A.1.210.3(1) | 2 |
| 3.A.2 | $H^+$- or $Na^+$-translocating F-type, V-type and A-type ATPase | F-ATPase | $H^+$, $Na^+$ | | Contig375gene9399 | 289 | 6 | 3.A.2.1.1(8) | 2 |
| | | | | | Contig375gene9400 | 88 | 0 | 3.A.2.1.1(8) | 3 |
| | | | | | Contig375gene9401 | 156 | 1 | 3.A.2.1.1(8) | 3 |
| | | | | | Contig375gene9402 | 180 | 0 | 3.A.2.1.1(8) | 2 |
| | | | | | Contig375gene9403 | 513 | 0 | 3.A.2.1.1(8) | 2 |
| | | | | | Contig375gene9404 | 291 | 0 | 3.A.2.1.1(8) | 2 |
| | | | | | Contig375gene9405 | 467 | 1 | 3.A.2.1.1(8) | 2 |
| | | | | 8 | Contig375gene9406 | 138 | 0 | 3.A.2.1.1(8) | 2 |
| 3.A.3 | P-type ATPase | P-ATPase | $Na^+$, $H^+$, $K^+$, $Ca^{2+}$, $Mg^{2+}$, $Cd^{2+}$, $Cu^{2+}$, $Zn^{2+}$, $Cd^{2+}$, $Co^{2+}$, $Ni^{2+}$, $Ag^+$, phospholipids (flipping) | | Contig373gene6510 | 920 | 10 | 3.A.3.2.4(1) | 2 |
| | | | | | Contig375gene9376 | 813 | 8 | 3.A.3.5.1(1) | 2 |
| | | | | | Contig369gene4263 | 805 | 8 | 3.A.3.5.5(1) | 2 |
| | | | | | Contig375gene7707 | 66 | 0 | 3.A.3.5.5(1) | 3 |
| | | | | | Contig375gene8429 | 752 | 0 | 3.A.3.5.7(1) | 3 |
| | | | | | Contig373gene6415 | 829 | 6 | 3.A.3.6.1(1) | 2 |
| | | | | | Contig374gene7074 | 794 | 6 | 3.A.3.6.4(1) | 2 |
| | | | | | Contig375gene8357 | 984 | 8 | 3.A.3.6.4(1) | 2 |
| | | | | | Contig373gene6441 | 799 | 8 | 3.A.3.6.4(1) | 2 |
| | | | | | Contig374gene7319 | 610 | 12 | 3.A.3.7.1(3) | 2 |
| | | | | | Contig374gene7320 | 743 | 7 | 3.A.3.7.1(3) | 2 |
| | | | | 12 | Contig374gene7321 | 203 | 1 | 3.A.3.7.1(3) | 2 |
| 3.A.5 | General secretory pathway | IISP | Proteins | | Contig363gene2920 | 463 | 0 | 3.A.5.1.1(11) | 2 |
| | | | | | Contig363gene2773 | 930 | 1 | 3.A.5.1.1(11) | 2 |
| | | | | | Contig374gene6838 | 948 | 0 | 3.A.5.1.1(11) | 2 |
| | | | | | Contig367gene3758 | 447 | 10 | 3.A.5.1.1(11) | 2 |
| | | | | 5 | Contig372gene5749 | 108 | 1 | 3.A.5.1.1(11) | 3 |
| 3.A.6 | Type III (virulence-related) secretory pathway | IIISP | Proteins | | Contig373gene6292 | 156 | 0 | 3.A.6.1.2(10) | 3 |

Copyright © 2005 John Wiley & Sons, Ltd.

| | | | | | | | | | |
|---|---|---|---|---|---|---|---|---|---|
| | | | | | Contig373gene6293 | 186 | 2 | 3.A.6.1.2(10) | 3 |
| | | | | | Contig373gene6294 | 264 | 4 | 3.A.6.1.2(10) | 2 |
| | | | | | Contig373gene6295 | 89 | 2 | 3.A.6.1.2(10) | 3 |
| | | | | | Contig373gene6296 | 253 | 6 | 3.A.6.1.2(10) | 2 |
| | | | | | Contig373gene6256 | 563 | 2 | 3.A.6.1.2(10) | 2 |
| | | | | | Contig373gene6258 | 278 | 0 | 3.A.6.1.2(10) | 2 |
| | | | | | Contig373gene6259 | 486 | 0 | 3.A.6.1.2(10) | 2 |
| | | | | | Contig371gene5351 | 380 | 4 | 3.A.6.1.2(10) | 2 |
| | | | | 10 | Contig371gene5352 | 695 | 8 | 3.A.6.1.2(10) | 2 |
| 3.A.7 | Type IV (conjugal DNA-protein transfer or VirB) secretory pathway | IVSP | Proteins, protein–DNA complexes | | Contig351gene1012 | 423 | 0 | 3.A.7.4.1(10) | 2 |
| | | | | | Contig342gene418 | 669 | 2 | 3.A.7.X | 2 |
| | | | | | Contig342gene420 | 358 | 0 | 3.A.7.4.1(10) | 2 |
| | | | | | Contig351gene1006 | 819 | 0 | 3.A.7.4.1(10) | 2 |
| | | | | | Contig351gene1007 | 245 | 1 | 3.A.7.4.1(10) | 3 |
| | | | | | Contig351gene1009 | 459 | 7 | 3.A.7.X | 3 |
| | | | | | Contig351gene1010 | 234 | 1 | 3.A.7.4.1(10) | 2 |
| | | | | | Contig351gene1011 | 330 | 0 | 3.A.7.4.1(10) | 2 |
| | | | | | Contig342gene423 | 809 | 0 | 3.A.7.4.1(10) | 2 |
| | | | | | Contig342gene424 | 241 | 1 | 3.A.7.4.1(10) | 3 |
| | | | | | Contig342gene427 | 234 | 0 | 3.A.7.4.1(10) | 3 |
| | | | | | Contig342gene428 | 333 | 0 | 3.A.7.4.1(10) | 2 |
| | | | | | Contig342gene429 | 422 | 1 | 3.A.7.4.1(10) | 2 |
| | | | | | Contig368gene4065 | 818 | 2 | 3.A.7.4.1(10) | 2 |
| | | | | | Contig368gene4066 | 252 | 1 | 3.A.7.4.1(10) | 3 |
| | | | | | Contig368gene4116 | 303 | 0 | 3.A.7.4.1(10) | 3 |
| | | | | | Contig368gene4117 | 414 | 2 | 3.A.7.4.1(10) | 2 |
| | | | | | Contig371gene5307 | 438 | 0 | 3.A.7.5.1(10) | 2 |
| | | | | | Contig365gene3342 | 456 | 0 | 3.A.7.5.1(10) | 2 |
| | | | | 20 | Contig368gene4062 | 349 | 0 | 3.A.7.5.1(10) | 2 |
| 3.A.11 | Bacterial competence-related DNA transformation transporter | DNA-T | Single-stranded DNA | 1 | Contig370gene5063 | 851 | 9 | 3.A.11.1.1(3) | 2 |
| 3.A.12 | Septal DNA translocator | S-DNA-T | DNA, DNA–protein complexes | | Contig357gene1647 | 1123 | 3 | 3.A.12.1.2(1) | 2 |
| | | | | 2 | Contig346gene649 | 775 | 4 | 3.A.12.1.2(1) | 2 |
| 3.A.13 | Filamentous phage exporter | FPhE | DNA | 1 | Contig374gene7161 | 358 | 0 | 3.A.13.1.1(1) | 2 |
| 3.A.15 | Outer membrane protein secreting main terminal branch | MTB | Pilin/fimbrilin | | Contig363gene2929 | 573 | 1 | 3.A.15.2.1(10) | 3 |
| | | | | | Contig363gene2930 | 421 | 4 | 3.A.15.2.1(10) | 2 |
| | | | | | Contig363gene2931 | 289 | 7 | 3.A.15.2.1(10) | 2 |
| | | | | | Contig346gene667 | 154 | 1 | 3.A.15.2.1(10) | 3 |
| | | | | | Contig372gene5842 | 347 | 0 | 3.A.15.2.1(10) | 2 |
| | | | | | Contig372gene5843 | 381 | 0 | 3.A.15.2.1(10) | 2 |
| | | | | | Contig375gene9231 | 202 | 1 | 3.A.15.X | 2 |

Copyright © 2005 John Wiley & Sons, Ltd.

**Table 3.** Continued

| Family (1) | Family (2) | Abbreviation (3) | Typical substrates (4) | Total # (5) | Gene (02jul03) (6) | Length (#aas) (7) | # TMSs (8) | Nearest homologue in TCDB (9) | Evidence (10) |
|---|---|---|---|---|---|---|---|---|---|
| | | | | | Contig375gene9232 | 442 | 3 | 3.A.15.X | 2 |
| | | | | | Contig375gene9233 | 568 | 0 | 3.A.15.X | 2 |
| | | | | | Contig352gene1091 | 182 | 1 | 3.A.15.2.1(10) | 3 |
| | | | | | Contig352gene1092 | 234 | 1 | 3.A.15.2.1(10) | 3 |
| | | | | | Contig374gene7449 | 147 | 2 | 3.A.15.2.1(10) | 3 |
| | | | | | Contig375gene8624 | 167 | 2 | 3.A.15.2.1(10) | 3 |
| | | | | | Contig368gene4125 | 635 | 0 | 3.A.15.2.1(10) | 3 |
| | | | | | Contig375gene7605 | 284 | 1 | 3.A.15.1.1(14) | 3 |
| | | | | | Contig375gene7606 | 327 | 1 | 3.A.15.1.1(14) | 3 |
| | | | | | Contig375gene7611 | 513 | 0 | 3.A.15.1.1(14) | 2 |
| | | | | 18 | Contig375gene7612 | 405 | 4 | 3.A.15.1.1(14) | 2 |
| 3.B. Decarboxylation-driven active transporters | | | | | | | | | |
| 3.B.1 | $Na^+$-transporting carboxylic acid decarboxylase | NaT-DC | $Na^+$ | | Contig358gene1826 | 539 | 4 | 3.B.1.1.2(5) | 2 |
| | | | | 2 | Contig365gene3364 | 535 | 3 | 3.B.1.1.2(5) | 2 |
| 3.D. Oxidoreduction-driven active transporters | | | | | | | | | |
| 3.D.1 | Proton-translocating NADH dehydrogenase | NDH | $H^+$ or $Na^+$ (efflux) | | Contig356gene1471 | 467 | 3 | 3.D.1.1.1(14) | 2 |
| | | | | | Contig370gene4970 | 119 | 3 | 3.D.1.2.1(14) | 2 |
| | | | | | Contig370gene4971 | 160 | 1 | 3.D.1.2.1(14) | 2 |
| | | | | | Contig370gene4972 | 199 | 0 | 3.D.1.2.1(14) | 2 |
| | | | | | Contig370gene4973 | 417 | 0 | 3.D.1.2.1(14) | 2 |
| | | | | | Contig370gene4974 | 168 | 1 | 3.D.1.2.1(14) | 3 |
| | | | | | Contig370gene4975 | 431 | 1 | 3.D.1.2.1(14) | 2 |
| | | | | | Contig370gene4976 | 828 | 1 | 3.D.1.2.1(14) | 2 |
| | | | | | Contig370gene4977 | 354 | 8 | 3.D.1.2.1(14) | 2 |
| | | | | | Contig370gene4979 | 163 | 0 | 3.D.1.2.1(14) | 2 |
| | | | | | Contig370gene4980 | 225 | 5 | 3.D.1.2.1(14) | 3 |
| | | | | | Contig370gene4981 | 101 | 3 | 3.D.1.2.1(14) | 2 |
| | | | | | Contig370gene4982 | 692 | 17 | 3.D.1.2.1(14) | 2 |
| | | | | | Contig370gene4984 | 491 | 14 | 3.D.1.2.1(14) | 2 |
| | | | | | Contig365gene3228 | 518 | 2 | 3.D.1.X | 2 |
| | | | | | Contig365gene3229 | 957 | 0 | 3.D.1.X | 2 |
| | | | | | Contig369gene4352 | 414 | 2 | 3.D.1.1.1(14) | 2 |
| | | | | | Contig364gene3085 | 402 | 1 | 3.D.1.1.1(14) | 3 |
| | | | | 19 | Contig370gene4983 | 488 | 14 | 3.D.1.3.1(14) | 2 |
| 3.D.2 | Proton-translocating transhydrogenase | PTH | $H^+$ (efflux) | | Contig334gene207 | 101 | 3 | 3.D.2.2.1(3) | 3 |
| | | | | | Contig334gene208 | 457 | 10 | 3.D.2.2.1(3) | 2 |
| | | | | | Contig326gene94 | 257 | 1 | 3.D.2.2.1(3) | 2 |
| | | | | | Contig372gene5764 | 401 | 0 | 3.D.2.2.1(3) | 2 |

Copyright © 2005 John Wiley & Sons, Ltd.

| | | | | | | | | | |
|---|---|---|---|---|---|---|---|---|---|
| | | | | | Contig372gene5765 | 152 | 3 | 3.D.2.2.1(3) | 2 |
| | | | | 6 | Contig372gene5766 | 490 | 10 | 3.D.2.2.1(3) | 2 |
| 3.D.3 | Proton-translocating quinol : cytochrome *c* reductase | QCR | $H^+$ (efflux) | | Contig367gene3822 | 205 | 1 | 3.D.3.1.1(3) | 2 |
| | | | | | Contig367gene3823 | 467 | 13 | 3.D.3.1.1(3) | 2 |
| | | | | 3 | Contig367gene3824 | 247 | 2 | 3.D.3.X | 3 |
| 3.D.4 | Proton-translocating cytochrome oxidase | COX | $H^+$ (efflux) | | Contig375gene8425 | 518 | 13 | 3.D.4.2.1(1) | 3 |
| | | | | | Contig360gene2255 | 482 | 12 | 3.D.4.3.1(1) | 3 |
| | | | | | Contig364gene3091 | 529 | 13 | 3.D.4.3.1(1) | 3 |
| | | | | | Contig370gene4992 | 322 | 3 | 3.D.4.5.1(5) | 2 |
| | | | | | Contig370gene4993 | 657 | 14 | 3.D.4.5.1(5) | 2 |
| | | | | | Contig370gene4994 | 214 | 5 | 3.D.4.5.1(5) | 2 |
| | | | | | Contig370gene4995 | 116 | 3 | 3.D.4.5.1(5) | 3 |
| | | | | | Contig374gene7516 | 308 | 9 | 3.D.4.5.1(5) | 2 |
| | | | | | Contig374gene7508 | 536 | 12 | 3.D.4.6.1(2) | 2 |
| | | | | | Contig374gene7512 | 286 | 7 | 3.D.4.X | 2 |
| | | | | | Contig375gene8971 | 391 | 3 | 3.D.4.X | 3 |
| | | | | | Contig375gene8972 | 585 | 12 | 3.D.4.X | 2 |
| | | | | | Contig375gene8973 | 222 | 5 | 3.D.4.X | 3 |
| | | | | | Contig375gene8974 | 234 | 5 | 3.D.4.X | 3 |
| | | | | | Contig372gene5641 | 319 | 3 | 3.D.4.5.1(5) | 2 |
| | | | | | Contig372gene5642 | 658 | 15 | 3.D.4.5.1(5) | 2 |
| | | | | | Contig372gene5643 | 226 | 5 | 3.D.4.5.1(5) | 2 |
| | | | | | Contig372gene5644 | 121 | 3 | 3.D.4.5.1(5) | 3 |
| | | | | | Contig375gene8913 | 349 | 4 | 3.D.4.5.1(5) | 2 |
| | | | | | Contig375gene8914 | 667 | 15 | 3.D.4.5.1(5) | 2 |
| | | | | | Contig375gene8915 | 218 | 5 | 3.D.4.5.1(5) | 2 |
| | | | | | Contig375gene8916 | 142 | 0 | 3.D.4.5.1(5) | 3 |
| | | | | 23 | Contig374gene7507 | 422 | 3 | 3.D.4.7.1(3) | 3 |
| 4. Phosphotransferase systems | | | | | | | | | |
| 4.A.6 | PTS mannose–fructose–sorbose | Man | Glucose, mannose, fructose, sorbose, etc. | | Contig362gene2525 | 316 | 3 | 4.A.6.1.1(3) | 3 |
| | | | | 2 | Contig374gene7493 | 151 | 0 | 4.A.6.1.2(4) | 3 |
| 5.A. Transmembrane electron transfer carriers | | | | | | | | | |
| 5.A.1 | Disulphide bond oxido-reductase D | DsbD | 2 $e^-$ | | Contig375gene8804 | 278 | 4 | 5.A.1.1.1(1) | 3 |
| | | | | 2 | Contig367gene3767 | 624 | 9 | 5.A.1.1.1(1) | 2 |
| 5.A.2 | Disulphide bond oxido-reductase B | DsbB | 2 $e^-$ | 1 | Contig336gene256 | 255 | 4 | 5.A.2.1.1(1) | 3 |
| 5.A.3 | Prokaryotic molybdopterin-containing oxidoreductase | PMO | Proton translocation | | Contig340gene359 | 701 | 0 | 5.A.3.2.1(3) | 3 |
| | | | | | Contig366gene3609 | 1025 | 1 | 5.A.3.2.1(3) | 2 |

Copyright © 2005 John Wiley & Sons, Ltd.

**Table 3.** Continued

| Family (1) | Family (2) | Abbreviation (3) | Typical substrates (4) | Total # (5) | Gene (02jul03) (6) | Length (#aas) (7) | # TMSs (8) | Nearest homologue in TCDB (9) | Evidence (10) |
|---|---|---|---|---|---|---|---|---|---|
| | | | | | Contig366gene3610 | 226 | 0 | 5.A.3.2.1(3) | 3 |
| | | | | | Contig366gene3612 | 418 | 6 | 5.A.3.2.1(3) | 2 |
| | | | | | Contig360gene2128 | 1252 | 0 | 5.A.3.1.1(3) | 2 |
| | | | | | Contig360gene2129 | 517 | 0 | 5.A.3.1.1(3) | 2 |
| | | | | 7 | Contig360gene2131 | 227 | 5 | 5.A.3.1.1(3) | 2 |
| 8.A. Auxiliary transport proteins | | | | | | | | | |
| 8.A.1 | Membrane fusion protein | MFP | Proteins, peptides, lipopolysaccharides, drugs, dyes, signalling molecules, heavy metal ions, etc. | | Contig358gene1816 | 378 | 1 | 8.A.1.1.1(1) | 2 |
| | | | | | Contig374gene7200 | 349 | 1 | 8.A.1.1.1(1) | 3 |
| | | | | | Contig360gene2101 | 413 | 2 | 8.A.1.1.1(1) | 2 |
| | | | | | Contig354gene1324 | 322 | 1 | 8.A.1.1.1(1) | 3 |
| | | | | | Contig375gene9176 | 328 | 1 | 8.A.1.1.1(1) | 3 |
| | | | | | Contig375gene8188 | 381 | 1 | 8.A.1.1.1(1) | 2 |
| | | | | | Contig375gene8550 | 380 | 2 | 8.A.1.1.1(1) | 2 |
| | | | | | Contig375gene8586 | 392 | 3 | 8.A.1.1.1(1) | 3 |
| | | | | | Contig364gene3066 | 423 | 2 | 8.A.1.1.1(1) | 2 |
| | | | | | Contig371gene5462 | 405 | 0 | 8.A.1.2.1(1) | 3 |
| | | | | | Contig373gene6080 | 505 | 1 | 8.A.1.2.1(1) | 2 |
| | | | | | Contig373gene6556 | 385 | 1 | 8.A.1.2.1(1) | 3 |
| | | | | | Contig373gene6562 | 404 | 1 | 8.A.1.2.1(1) | 3 |
| | | | | | Contig375gene8616 | 520 | 0 | 8.A.1.2.1(1) | 1 |
| | | | | | Contig361gene2415 | 523 | 0 | 8.A.1.2.1(1) | 3 |
| | | | | | Contig363gene2862 | 407 | 1 | 8.A.1.2.1(1) | 3 |
| | | | | | Contig369gene4235 | 93 | 1 | 8.A.1.2.1(1) | 3 |
| | | | | | Contig369gene4236 | 292 | 0 | 8.A.1.2.1(1) | 1 |
| | | | | | Contig368gene3998 | 395 | 1 | 8.A.1.2.1(1) | 1 |
| | | | | | Contig329gene132 | 387 | 1 | 8.A.1.6.1(1) | 2 |
| | | | | | Contig353gene1238 | 387 | 0 | 8.A.1.6.1(1) | 3 |
| | | | | | Contig358gene1807 | 412 | 0 | 8.A.1.6.1(1) | 3 |
| | | | | | Contig353gene1180 | 398 | 2 | 8.A.1.6.1(1) | 2 |
| | | | | | Contig375gene7758 | 407 | 3 | 8.A.1.6.1(1) | 2 |
| | | | | 25 | Contig375gene7764 | 415 | 0 | 8.A.1.6.1(1) | 2 |
| 8.A.3 | Cytoplasmic membrane-periplasmic auxiliary-1 (MPA1) protein with cytoplasmic (C) domain | MPA1 | Complex polysaccharides | | Contig366gene3603 | 362 | 0 | 8.A.3.2.2(2) | 3 |
| | | | | | Contig372gene5596 | 748 | 1 | 8.A.3.3.1(1) | 2 |
| | | | | 3 | Contig372gene5968 | 777 | 2 | 8.A.3.3.2(1) | 2 |

Copyright © 2005 John Wiley & Sons, Ltd.

| | | | | | | | | | |
|---|---|---|---|---|---|---|---|---|---|
| 8.A.4 | Cytoplasmic membrane-periplasmic auxiliary-2 | MPA2 | Complex polysaccharides | 1 | Contig375gene8671 | 368 | 2 | 8.A.4.1.1(1) | 2 |
| 8.A.7 | Phosphotransferase system enzyme I | EI | Sugars | 1 | Contig374gene7495 | 585 | 0 | 8.A.7.1.1(1) | 2 |
| 8.A.8 | Phosphotransferase system HPr | HPr | Sugars | 1 | Contig374gene7494 | 89 | 1 | 8.A.8.1.1(1) | 3 |
| 9.A. Transporters of unknown classification | | | | | | | | | |
| 9.A.2 | MerTP mercuric ion ($Hg^{2+}$) permease | MerTP | $Hg^{2+}$ (uptake) | | Contig375gene8504 | 88 | 0 | 9.A.2.1.1(1) | 3 |
| | | | | | Contig375gene8370 | 95 | 0 | 9.A.2.1.1(1) | 3 |
| | | | | 3 | Contig369gene4509 | 91 | 0 | 9.A.2.1.1(1) | 2 |
| 9.A.8 | Ferrous iron uptake | FeoB | $Fe^{2+}$ (uptake) | 1 | Contig372gene5560 | 620 | 11 | 9.A.8.1.1(1) | 2 |
| 9.A.10 | Oxidase-dependent $Fe^{2+}$ transporter | OFeT | $Fe^{2+}$ (uptake) | | Contig375gene9242 | 504 | 1 | 9.A.10.1.1(2) | 3 |
| | | | | | Contig369gene4470 | 614 | 1 | 9.A.10.1.1(2) | 3 |
| | | | | 3 | Contig375gene8124 | 605 | 1 | 9.A.10.1.1(2) | 3 |
| 9.A.17 | Lead | PbrT | Lead resistance | | Contig373gene6437 | 642 | 7 | 9.A.17.1.1(1) | 2 |
| | | | | 2 | Contig369gene4270 | 254 | 1 | 9.A.17.1.1(1) | 2 |
| 9.A.21 | ComC DNA uptake competence | ComC | DNA, proteins | 1 | Contig375gene8629 | 1102 | 1 | 9.A.21.1.1(1) | 3 |
| 9.B. Putative uncharacterized transporters | | | | | | | | | |
| 9.B.3 | Putative bacterial murein precursor exporter | MPE | Lipid-linked murein precursors such as NAG-NAM-pentapeptide pyrophosphoryl undecaprenol (lipid II) | | Contig363gene2762 | 413 | 9 | 9.B.3.1.1(1) | 2 |
| | | | | 2 | Contig374gene7332 | 380 | 9 | 9.B.3.1.2(1) | 2 |
| 9.B.4 | Putative efflux transporter | PET | Unknown | | Contig367gene3857 | 790 | 12 | 9.B.4.1.1(1) | 2 |
| | | | | | Contig353gene1242 | 664 | 11 | 9.B.4.1.2(1) | 3 |
| | | | | | Contig354gene1321 | 728 | 12 | 9.B.4.1.2(1) | 3 |
| | | | | 4 | Contig375gene9174 | 659 | 11 | 9.B.4.1.2(1) | 2 |
| 9.B.10 | 6 TMS putative MarC transporter | MarC | Multiple antibiotic resistance | 1 | Contig367gene3809 | 207 | 6 | 9.B.10.1.1(1) | 3 |
| 9.B.14 | Putative heme exporter protein | HEP | Heme | | Contig375gene8799 | 653 | 15 | 9.B.14.1.1(1) | 2 |
| | | | | 2 | Contig357gene1726 | 680 | 15 | 9.B.14.1.1(1) | 2 |
| 9.B.17 | Putative fatty acid transporter | FAT | Fatty acyl CoA ligases (fatty acyl CoA synthases), carnitine CoA ligases, and putative transporters | | Contig349gene843 | 549 | 4 | 9.B.17.1.4(1) | 2 |
| | | | | | Contig353gene1184 | 617 | 2 | 9.B.17.1.4(1) | 2 |
| | | | | | Contig340gene362 | 560 | 1 | 9.B.17.1.4(1) | 2 |
| | | | | | Contig362gene2701 | 629 | 2 | 9.B.17.1.4(1) | 2 |
| | | | | | Contig362gene2706 | 553 | 0 | 9.B.17.1.4(1) | 2 |
| | | | | | Contig373gene6071 | 631 | 0 | 9.B.17.1.4(1) | 3 |
| | | | | | Contig358gene1823 | 548 | 1 | 9.B.17.1.4(1) | 2 |

Copyright © 2005 John Wiley & Sons, Ltd.

**Table 3.** Continued

| Family (1) | Family (2) | Abbreviation (3) | Typical substrates (4) | Total # (5) | Gene (02jul03) (6) | Length (#aas) (7) | # TMSs (8) | Nearest homologue in TCDB (9) | Evidence (10) |
|---|---|---|---|---|---|---|---|---|---|
| | | | | | Contig355gene1404 | 516 | 1 | 9.B.17.1.4(1) | 2 |
| | | | | | Contig374gene7145 | 564 | 0 | 9.B.17.1.4(1) | 2 |
| | | | | | Contig345gene613 | 555 | 2 | 9.B.17.1.4(1) | 2 |
| | | | | | Contig366gene3582 | 517 | 0 | 9.B.17.1.4(1) | 2 |
| | | | | | Contig375gene8255 | 630 | 0 | 9.B.17.1.4(1) | 2 |
| | | | | | Contig366gene3706 | 566 | 0 | 9.B.17.1.4(1) | 2 |
| | | | | | Contig373gene6519 | 510 | 0 | 9.B.17.1.4(1) | 2 |
| | | | | | Contig373gene6406 | 515 | 0 | 9.B.17.1.4(1) | 2 |
| | | | | | Contig375gene8238 | 509 | 0 | 9.B.17.1.4(1) | 2 |
| | | | | | Contig367gene3834 | 500 | 0 | 9.B.17.1.4(1) | 2 |
| | | | | | Contig371gene5171 | 545 | 0 | 9.B.17.1.4(1) | 2 |
| | | | | | Contig371gene5425 | 523 | 0 | 9.B.17.1.4(1) | 2 |
| | | | | | Contig371gene5430 | 510 | 3 | 9.B.17.1.4(1) | 2 |
| | | | | | Contig371gene5447 | 501 | 0 | 9.B.17.1.4(1) | 2 |
| | | | | | Contig365gene3363 | 570 | 0 | 9.B.17.1.4(1) | 2 |
| | | | | | Contig373gene6687 | 517 | 0 | 9.B.17.1.4(1) | 2 |
| | | | | | Contig372gene5610 | 518 | 0 | 9.B.17.1.4(1) | 2 |
| | | | | | Contig375gene8757 | 660 | 0 | 9.B.17.1.6(1) | 2 |
| | | | | | Contig370gene4532 | 626 | 1 | 9.B.17.1.6(1) | 2 |
| | | | | | Contig373gene6475 | 545 | 1 | 9.B.17.1.6(1) | 2 |
| | | | | | Contig371gene5414 | 567 | 2 | 9.B.17.1.6(1) | 3 |
| | | | | | Contig373gene6157 | 527 | 1 | 9.B.17.1.6(1) | 2 |
| | | | | 30 | Contig373gene6682 | 525 | 1 | 9.B.17.1.6(1) | 2 |
| 9.B.20 | Putative $Mg^{2+}$ transporter-C | MgtC | $Mg^{2+}$ | | Contig375gene8497 | 152 | 4 | 9.B.20.2.1(1) | 3 |
| | | | | 2 | Contig385gene222 | 240 | 4 | | |
| 9.B.22 | Putative permease | PerM | Unknown | 1 | Contig357gene1729 | 361 | 7 | 9.B.22.1.3(1) | 3 |
| 9.B.24 | Testis-enhanced gene transfer | TEGT | Glucose (and fructose?) uptake or metabolism, cell death | 1 | Contig360gene2157 | 235 | 7 | 9.B.24.2.1(1) | 2 |
| 9.B.26 | PC-terminal fragment 7 | PC-terminal fragment 7 | Unknown | 1 | Contig370gene4655 | 241 | 5 | 9.B.26.1.1(1) | 3 |
| 9.B.30 | Hly III | Hly III | Unknown | 1 | Contig375gene8924 | 205 | 7 | 9.B.30.1.1(1) | 2 |
| 9.B.32 | Putative vectorial glycosyl polymerization | VGP | Polysaccharides | | Contig353gene1189 | 658 | 7 | 9.B.32.1.3(1) | 3 |
| | | | | 2 | Contig369gene4253 | 367 | 2 | 9.B.32.1.3(1) | 3 |
| 9.B.37 | HlyC/CorC | HCC | Ions? | | Contig353gene1170 | 530 | 7 | 9.B.37.1.2(1) | 3 |
| | | | | | Contig346gene665 | 438 | 5 | 9.B.37.2.1(1) | 2 |
| | | | | 3 | Contig375gene7878 | 437 | 3 | 9.B.37.2.1(1) | 2 |
| 9.B.40 | DotA/TraY | DotA/TraY | Unknown | 1 | Contig366gene3548 | 751 | 7 | 9.B.40.1.2(1) | 2 |
| 9.B.42 | ExeAB | ExeAB | Secretin | 1 | Contig375gene8813 | 277 | 0 | 9.B.42.1.1(2) | 3 |
| 9.B.43 | YedZ | YedZ | Unknown | 1 | Contig367gene3778 | 224 | 6 | 9.B.43.1.1(1) | 2 |

Copyright © 2005 John Wiley & Sons, Ltd. 

| | | | | | | | | | |
|---|---|---|---|---|---|---|---|---|---|
| 9.B.45 | YnfA | YnfA | Unknown | 1 | Contig375gene8050 | 105 | 4 | 9.B.45.1.1(1) | 2 |
| 9.B.53 | Unknown IT-6 | UIT6 | Unknown | 1 | Contig347gene696 | 476 | 12 | 9.B.53.1.1(0) | 3 |
| Unclassified | | | | | | | | | |
| | Unclassified | Unclassified | Unknown | | Contig358gene1867 | 397 | 6 | N/A(0) | 3 |
| | | | | | Contig353gene1240 | 384 | 4 | N/A(0) | 3 |
| | | | | | Contig370gene4727 | 380 | 0 | N/A(0) | 3 |
| | | | | | Contig370gene4852 | 195 | 0 | N/A(0) | 3 |
| | | | | | Contig355gene1383 | 409 | 10 | N/A(0) | 3 |
| | | | | | Contig366gene3534 | 372 | 6 | N/A(0) | 3 |
| | | | | | Contig366gene3535 | 387 | 6 | N/A(0) | 3 |
| | | | | | Contig375gene7560 | 363 | 9 | N/A(0) | 3 |
| | | | | | Contig358gene1814 | 388 | 8 | N/A(0) | 3 |
| | | | | | Contig375gene8797 | 63 | 1 | N/A(0) | 3 |
| | | | | | Contig365gene3385 | 367 | 7 | N/A(0) | 3 |
| | | | | | Contig342gene421 | 127 | 0 | N/A(0) | 3 |
| | | | | | Contig342gene426 | 461 | 9 | N/A(0) | 3 |
| | | | | | Contig342gene428 | 333 | 0 | N/A(0) | 3 |
| | | | | | Contig373gene6385 | 3750 | 1 | N/A(0) | 3 |
| | | | | | Contig367gene3796 | 258 | 6 | N/A(0) | 3 |
| | | | | | Contig367gene3797 | 179 | 1 | N/A(0) | 3 |
| | | | | | Contig375gene9280 | 83 | 0 | N/A(0) | 3 |
| | | | | | Contig372gene5736 | 241 | 6 | N/A(0) | 3 |
| | | | | | Contig362gene2646 | 389 | 6 | N/A(0) | 3 |
| | | | | | Contig371gene5477 | 273 | 1 | N/A(0) | 3 |
| | | | | | Contig371gene5133 | 335 | 1 | N/A(0) | 3 |
| | | | | | Contig375gene9091 | 273 | 6 | N/A(0) | 3 |
| | | | | | Contig375gene8503 | 115 | 3 | N/A(0) | 3 |
| | | | | | Contig374gene6814 | 955 | 0 | N/A(0) | 3 |
| | | | | | Contig374gene6977 | 376 | 6 | N/A(0) | 3 |
| | | | | | Contig374gene7342 | 316 | 10 | N/A(0) | 3 |
| | | | | | Contig365gene3237 | 268 | 1 | N/A(0) | 3 |
| | | | | | Contig356gene1538 | 232 | 7 | N/A(0) | 3 |
| | | | | | Contig352gene1090 | 231 | 7 | N/A(0) | 3 |
| | | | | | Contig352gene1095 | 336 | 0 | N/A(0) | 3 |
| | | | | | Contig375gene8369 | 116 | 3 | N/A(0) | 3 |
| | | | | | Contig375gene8372 | 562 | 0 | | 3 |
| | | | | | Contig364gene3037 | 253 | 7 | N/A(0) | 3 |
| | | | | | Contig372gene5975 | 467 | 12 | N/A(0) | 3 |
| | | | | | Contig367gene3796 | 258 | 6 | N/A(0) | 3 |
| | | | | | Contig375gene7603 | 174 | 1 | N/A(0) | 3 |
| | | | | | Contig375gene7604 | 134 | 1 | N/A(0) | 3 |

Copyright © 2005 John Wiley & Sons, Ltd.

**Table 3.** Continued

| Family (1) | Family (2) | Abbreviation (3) | Typical substrates (4) | Total # (5) | Gene (02jul03) (6) | Length (#aas) (7) | # TMSs (8) | Nearest homologue in TCDB (9) | Evidence (10) |
|---|---|---|---|---|---|---|---|---|---|
| | | | | | Contig375gene7607 | 509 | 0 | N/A(0) | 3 |
| | | | | | Contig375gene7608 | 188 | 1 | N/A(0) | 3 |
| | | | | | Contig375gene7609 | 268 | 1 | N/A(0) | 3 |
| | | | | | Contig369gene4471 | 427 | 0 | N/A(0) | 3 |
| | | | | | Contig369gene4472 | 132 | 1 | N/A(0) | 3 |
| | | | | | Contig369gene4473 | 305 | 8 | N/A(0) | 3 |
| | | | | | Contig369gene4474 | 158 | 0 | N/A(0) | 3 |
| | | | | | Contig369gene4481 | 435 | 0 | N/A(0) | 3 |
| | | | | | Contig375gene9429 | 402 | 12 | N/A(0) | 3 |
| | | | | | Contig375gene8485 | 366 | 0 | N/A(0) | 3 |
| | | | | | Contig375gene8120 | 419 | 1 | N/A(0) | 3 |
| | | | | | Contig375gene8125 | 360 | 1 | N/A(0) | 3 |
| | | | | | Contig375gene8126 | 128 | 0 | N/A(0) | 3 |
| | | | | | Contig369gene4508 | 116 | 3 | N/A(0) | 3 |
| | | | | | Contig368gene4000 | 351 | 10 | N/A(0) | 3 |
| | | | | | Contig368gene4195 | 324 | 0 | N/A(0) | 3 |
| | | | | 55 | Contig368gene4197 | 197 | 0 | N/A(0) | 3 |
| | | | | 932 | | | | | |

[a] A full version of the table containing all the various names of the CH34 genes is provided as on-line supplementary material at: **http://bionomie.mikrobiologie.uni-halle.de/SupMat/Roz_05/Table 3.htm**

Copyright © 2005 John Wiley & Sons, Ltd.

structure is established for several members of this family. Three members of the OmpA-OmpF porin (OOP) family and a single FadL homologue, presumably concerned with transport of fatty acids across the outer membrane, were identified.

The next two families listed in Table 3, the FUP and AT families, with three members and one member, respectively, are concerned with export of proteins across the outer membrane. The three FUP ushers probably export fimbrial subunits for the assembly of 3 structurally and functionally distinct fimbriae. AT family members export their own N-terminal domains, which in this case may be a large cell surface protein. However, no surface layer could be observed for Rme (D. Neumann and D. H. Nies, unpublished data).

Seventeen OMR family members were identified. Fifteen of these are probably concerned with uptake of iron siderophore complexes (subfamilies 1 and 9). One is probably the Rme vitamin $B_{12}$ porin (subfamily 3). The single member of subfamily 4 may be concerned with copper acquisition.

Outer membrane factors (OMFs; TC #1.B.17) generally mediate efflux of heavy metals, drugs and macromolecules across the outer membrane in conjunction with an active efflux pump in the inner membrane. Twenty-eight homologues were identified. Of these, one is in subfamily 1 (a general OMF able to interact with multiple efflux pumps), eight are in subfamily 2 (concerned with heavy metal ion efflux), and 19 are in subfamily 3 (concerned with export of macromolecules, drugs and metals). Two members of this last subfamily resemble oligosaccharide exporters; four most resemble protein exporters; seven may be involved in export of drugs and other hydrophobic substances; and three may function in copper ion efflux.

Two members of the OMA family (1.B.18) are presumed to function in exopolysaccharide export, one member of the OprB family (1.B.19) probably allows facilitation of small molecules across the outer membrane, and the two members of the TPS family (1.B.20) most likely export proteins. Most of the six secretins (1.B.22) also probably function in protein export. Finally, the two OmpW family members (1.B.39) may export drugs and other hydrophobic molecules.

A channel-forming colicin-like protein (1.C.1), resembling colicin A of *Citrobacter freundii*, was found. A single holin (1.E.14), presumably involved in autolysin export for the purpose of promoting cell death, is also present.

### Secondary carriers

By far the largest number secondary carriers encoded within the Rme genome are members of the major facilitator superfamily (MFS). Rme has 83 recognizable MFS carriers. As shown in Table 3 and summarized in Table 4, 32 of these MFS permeases are putative drug/amphiphile/hydrophobe transporters of MFS families DHA1 (16 members), DHA2 (15 members) and DHA3 (1 member) (Busch and Saier, 2002). Some of these are likely to serve as lipid exporters, but others undoubtedly play primary roles in defence, in toxic substance export or in metabolite export.

Just one sugar transporter (SP family), one organophosphate porter (OPA family), 15 metabolite transporters (MHS family), three nitrate/nitrite transporters (NNP family), and three oxalate : formate antiporters (OFA) of the MFS allow uptake of essential nutrients. Additionally, one SHS porter, nine ACS porters, five AAHS porters, and one CP porter all probably function to bring organoanions into the cell. The OCT porter may transport organocations. Other MFS paralogues represented, with usually a single protein member in any one family, undoubtedly transport a wide range of other substances (Table 3).

Six amino acid/polyamine/organocation (APC) superfamily members were identified. Two of the subfamilies in the APC superfamily are represented. These porters are predicted to transport a range of zwitterionic and basic amino acids.

The CDF family and the ZIP family of heavy metal divalent cation transporters are represented with three and one members, respectively. All three CDF proteins have been characterized in detail (Anton *et al.*, 2004; Munkelt *et al.*, 2004). They belong to different clusters of the CDF protein family (Nies, 2003) and transport $Cd^{2+}$, $Co^{2+}$, $Zn^{2+}$, $Fe^{2+}$ and $Ni^{2+}$. A single member of the NiCoT family (TC #2.A.53), probably a $Ni^{2+}$ transporter, was also identified. A related protein is involved in nickel uptake for synthesis of the hydrogenases in the related bacterium *Ralstonia eutropha* (Degen and Eitinger, 2002; Eberz *et al.*, 1989; Eitinger and Friedrich, 1991, 1994; Eitinger *et al.*, 1997; Wolfram *et al.*, 1991, 1995).

Copyright © 2005 John Wiley & Sons, Ltd. 

**Table 4.** Family associations including subfamilies within the MFS, APC, RND, DMT, MOP and ABC superfamilies of transporter constituents

| Family | Abbreviation | Typical substrates | No. of members (%) |
|---|---|---|---|
| 1.A.1 | VIC | $Na^+$, $K^+$, $Ca^{2+}$, multiple cations | 2 (0.2) |
| 1.A.8 | MIP | $H_2O$, glycerol, urea, polyols, $NH_3$, $CO_2$ | 2 (0.2) |
| 1.A.11 | ClC | $Cl^-$, anions | 4 (0.4) |
| 1.A.20 | CytB | $H^+$ | 1 (0.1) |
| 1.A.22 | MscL | Proteins, ions (slightly cation-selective) | 1 (0.1) |
| 1.A.23 | MscS | Ions (slight anion selectivity) | 9 (1) |
| 1.A.30 | Mot/Exb-Mot | $H^+$, $Na^+$ | 2 (0.2) |
| 1.A.33 | Hsp70 | Ions, polypeptides | 2 (0.2) |
| 1.A.35 | MIT | Heavy-metal ions, $Mg^{2+}$, $Mn^{2+}$, $Co^{2+}$, $Ni^{2+}$, $Fe^{2+}$, $Al^{3+}$, $Mn^{2+}$ | 4 (0.4) |
| 1.B.1 | GBP | Ions, small ($M_r$ < 1000 Da) molecules | 29 (3.1) |
| 1.B.6 | OOP | Ions, small molecules | 3 (0.3) |
| 1.B.9 | FadL | Fatty acid, toluene, *m*-xylene and benzyl alcohol | 1 (0.1) |
| 1.B.11 | FUP | Protein folding and subunit assembly | 3 (0.3) |
| 1.B.12 | AT | N-terminal protein domains | 1 (0.1) |
| 1.B.14 | OMR | Iron–siderophore complexes, vitamin $B_{12}$, $Cu^{2+}$, colicin, DNA of various phages | 17 (1.8) |
| 1.B.17 | OMF | Heavy metal cations, drugs, oligosaccharides, proteins, etc. | 28 (3) |
| 1.B.18 | OMA | Exo- or capsular polysaccharide | 2 (0.2) |
| 1.B.19 | OprB | Ions, small molecules | 1 (0.1) |
| 1.B.20 | TPS | Proteins | 2 (0.2) |
| 1.B.22 | Secretin | Proteins | 6 (0.6) |
| 1.B.39 | OmpW | Methyl viologen and benzyl viologen | 2 (0.2) |
| 1.C.1 | Colicin | Ions, small molecules | 1 (0.1) |
| 1.E.14 | LrgA Holin | $Zn^{2+}$, $Fe^{2+}$ | 1 (0.1) |
| 2.A.1 | MFS | Various small molecules | Total 83 (8.9) |
| | -SP (1) | Sugars | 1 (0.1) |
| | -DHA1 (12 spanner) (2) drugs | Drugs | 16 (1.7) |
| | -DHA2 (14 spanner) (3) drugs | Drugs | 15 (1.6) |
| | -OPA (4) | Sugars, glycerol | 1 (0.1) |
| | -MHS (6) | Dicarboxylates, tricarboxylates | 15 (1.6) |
| | -NNP (8) | Nitrate, nitrite | 3 (0.3) |
| | -OFA (11) | Oxalate, formate | 3 (0.3) |
| | -SHS (12) | Sialate, lactate, pyruvate | 1 (0.1) |
| | -ACS (14) | Organic acids | 9 (1) |
| | -AAHS (15) | Aromatic acids | 5 (0.5) |
| | -CP (17) | Cyanate | 1 (0.1) |
| | -OCT (19) | Organic cations | 1 (0.1) |
| | -SET (20) | Sugars | 1 (0.1) |
| | -DHA3 (12 spanner) (21) drugs | Drugs | 1 (0.1) |
| | -VNT (22) | Neurotransmitter | 1 (0.1) |
| | -BST (23) | Unknown | 1 (0.1) |
| | -PAT (25) | Peptides, AcCoA | 1 (0.1) |
| | -UMC-terminal fragment (26) | Unknown | 1 (0.1) |
| | -PPP (27) | Phenylpropionate | 1 (0.1) |
| | -ADT (30) | Abietane diterpenoid | 1 (0.1) |
| | -Nre (31) | $Ni^{2+}$ | 1 (0.1) |
| | -Fsr (35) | Fosmidomycin | 1 (0.1) |
| | -AtoE (37) | Short chain fatty | 2 (0.2) |
| 2.A.3 | APC | Amino acids, polyamines, choline | Total 6 (0.6) |
| | -AAA (1) | Amino acids | 5 (0.5) |
| | -CAT (3) | Cationic amino acids | 1 (0.1) |
| 2.A.4 | CDF | $Cd^{2+}$, $Co^{2+}$, $Zn^{2+}$ | 3 (0.3) |
| 2.A.5 | ZIP | $Zn^{2+}$, $Fe^{2+}$ | 1 (0.1) |

Copyright © 2005 John Wiley & Sons, Ltd. 

**Table 4.** Continued

| Family | Abbreviation | Typical substrates | No. of members (%) |
|---|---|---|---|
| 2.A.6 | RND | Heavy metal ions, multiple drugs, oligosaccharides, organic solvents, fatty acids, phospholipids, cholesterol | Total 30 (3.2) |
| | -HME (1) | Heavy metals | 17 (1.8) |
| | -HAE1 (2) | Hydrophobe/amphiphiles | 9 (1) |
| | -SecDF(4) | Sec secretory accessory proteins | 2 (0.2) |
| | -HAE2 (5) | Hydrophobe/amphiphiles | 1 (0.1) |
| | -ORF4 (8) | Hydrophobe/amphiphiles | 1 (0.1) |
| 2.A.7 | DMT | Multiple drugs and dyes (mostly cationic) | Total 18 (1.9) |
| | -SMR (1) | Drugs | 2 (0.2) |
| | -BAT (2) | Unknown | 2 (0.2) |
| | -DME (3) | Drugs, metabolites | 12 (1.3) |
| | -RarD (7) | Chloramphenicol | 2 (0.2) |
| 2.A.9 | Oxa1 | Proteins | 1 (0.1) |
| 2.A.10 | KDGT | 2-Keto-3-deoxygluconate | 1 (0.1) |
| 2.A.11 | CitMHS | Citrate | 1 (0.1) |
| 2.A.12 | AAA | ATP, ADP | 1 (0.1) |
| 2.A.14 | LctP | Lactate | 1 (0.1) |
| 2.A.19 | CaCA | $Ca^{2+}$ | 1 (0.1) |
| 2.A.20 | PiT | Inorganic phosphate | 1 (0.1) |
| 2.A.21 | SSS | Sugars, amino acids, vitamins, nucleosides, inositols, iodide, urea | 5 (0.5) |
| 2.A.23 | DAACS | $C_4$-dicarboxylates, acidic and neutral amino acids | 5 (0.5) |
| 2.A.24 | CCS | Mono-, di-, and tricarboxylates | 1 (0.1) |
| 2.A.36 | CPA1 | $Na^+/H^+$, $Na^+$ or $K^+/H^+$ | 1 (0.1) |
| 2.A.37 | CPA2 | $Na^+/H^+$ or $K^+/H^+$ | 6 (0.6) |
| 2.A.40 | NCS2 | Nucleobases, urate | 3 (0.3) |
| 2.A.45 | ArsB | Arsenite, antimonite | 1 (0.1) |
| 2.A.46 | BenE | Benzoate | 1 (0.1) |
| 2.A.47 | DASS | Dicarboxylates, phosphate, sulphate | 4 (0.4) |
| 2.A.49 | Amt | Ammonium | 2 (0.2) |
| 2.A.51 | CHR | Chromate, sulphate (uptake or efflux) | 4 (0.4) |
| 2.A.52 | NiCoT | $Ni^{2+}$, $Co^{2+}$ | 1 (0.1) |
| 2.A.53 | SulP | Sulphate | 5 (0.5) |
| 2.A.56 | TRAP−T | $C_4$-dicarboxylates, acidic amino acids, sugars? | 6 (0.6) |
| 2.A.58 | PNaS | Inorganic phosphate | 2 (0.2) |
| 2.A.59 | ACR3 | Arsenite | 1 (0.1) |
| 2.A.64 | Tat | Redox proteins | 4 (0.4) |
| 2.A.66 | MOP | Drugs, lipid-linked oligosaccharide precursors | Total 5 (0.5) |
| | -MATE (1) | Drugs | 3 (0.3) |
| | -PST (2) | Polysaccharides | 1 (0.1) |
| | -MVF (4) | Unknown | 1 (0.1) |
| 2.A.67 | OPT | Peptides | 2 (0.2) |
| 2.A.69 | AEC | Auxin (efflux) | 2 (0.2) |
| 2.A.72 | KUP | $K^+$ (uptake) | 1 (0.1) |
| 2.A.75 | LysE | Basic amino acids | 1 (0.1) |
| 2.A.76 | RhtB | Neutral amino acids and their derivatives | 11 (1.2) |
| 2.A.78 | LIV−E | Carboxylates, amino acids, amines (efflux) | 1 (0.1) |
| 2.A.80 | TTT | Tricarboxylate | 74 (8) |
| 2.A.81 | AAE | Aspartate, alanine | 2 (0.2) |
| 2.C.1 | TonB | $H^+$?, drives solute uptake across outer bacterial membranes | 5 (0.5) |
| 3.A.1 | ABC | All sorts of inorganic and organic molecules of small, intermediate, and large sizes, from simple ions to macromolecules | Total 213 (23) |
| | -CUT1(1) | Sugars, metabolites | 15 (1.6) |

Copyright © 2005 John Wiley & Sons, Ltd. 

**Table 4.** Continued

| Family | Abbreviation | Typical substrates | No. of members (%) |
|---|---|---|---|
| | -CUT2 (2) | Sugars, metabolites | 4 (0.4) |
| | -PAAT (3) | Polar amino acids | 14 (1.5) |
| | -HAAT (4) | Hydrophobic amino acids | 45 (4.8) |
| | -PepT (5) | Peptide, opine, nickel | 33 (3.5) |
| | -SulT (6) | Sulphate, tungstate | 6 (0.6) |
| | -PhoT (7) | Phosphate | 6 (0.6) |
| | -MolT (8) | Molybdate | 4 (0.4) |
| | -PhnT (9) | Phosphonate | 6 (0.6) |
| | -POPT(11) | Polyamine, opine, phosphonate | 8 (0.9) |
| | -QAT (12) | Quaternary amine | 5 (0.5) |
| | -VB12T(13) | Vitamin $B_{12}$ | 1 (0.1) |
| | -FeCT (14) | Iron chelate | 5 (0.5) |
| | -MZT (15) | Manganese, zinc, iron chelate | 1 (0.1) |
| | -NitT (16) | Nitrate, nitrite, cyanate | 14 (1.5) |
| | -TauT (17) | Taurine | 13 (1.4) |
| | -BIT (20) | $Fe^{3+}$ | 1 (0.1) |
| | -CPSE (101) | Capsular polysaccharides | 2 (0.2) |
| | -LOSE (102) | Lipo-oligosaccharide | 7 (0.8) |
| | -DrugE1(105) | Drugs | 2 (0.2) |
| | -HemeE(107) | Heme | 6 (0.6) |
| | -Prot1E (109) | Proteins | 1 (0.1) |
| | -Prot2E (110) | Proteins | 1 (0.1) |
| | -Drug RA1 (120) | Drugs | 5 (0.5) |
| | -Drug RA2 (121) | Drugs | 1 (0.1) |
| | -MacB (122) | Macrolide | 2 (0.2) |
| | -LPT (125) | Lipoproteins | 3 (0.3) |
| | -HMT (210) | Heavy metals | 2 (0.2) |
| 3.A.2 | F-ATPase | $H^+$, $Na^+$ | 8 (0.9) |
| 3.A.3 | P-ATPase | $Na^+$, $H^+$, $K^+$, $Ca^{2+}$, $Mg^{2+}$, $Cd^{2+}$, $Cu^{2+}$, $Zn^{2+}$, $Cd^{2+}$, $Co^{2+}$, $Ni^{2+}$, $Ag^+$, phospholipids (flipping) | 12 (1.3) |
| 3.A.5 | IISP | Proteins | 5 (0.5) |
| 3.A.6 | IIISP | Proteins | 10 (1.1) |
| 3.A.7 | IVSP | Proteins, protein–DNA complexes | 20 (2.2) |
| 3.A.11 | DNA-T | Single-stranded DNA | 1 (0.1) |
| 3.A.12 | S-DNA-T | DNA, DNA–protein complexes | 2 (0.2) |
| 3.A.13 | FPhE | Viruses | 1 (0.1) |
| 3.A.15 | MTB | Pilin/fimbrilin | 18 (1.9) |
| 3.B.1 | NaT-DC | $Na^+$ | 2 (0.2) |
| 3.D.1 | NDH | $H^+$ or $Na^+$ (efflux) | 19 (2) |
| 3.D.2 | PTH | $H^+$ (efflux) | 6 (0.6) |
| 3.D.3 | QCR | $H^+$ (efflux) | 3 (0.3) |
| 3.D.4 | COX | $H^+$ (efflux) | 23 (2.5) |
| 4.A.6 | Man | Glucose, mannose, fructose, sorbose, etc. | 2 (0.2) |
| 5.A.1 | DsbD | 2 $e^-$ | 2 (0.2) |
| 5.A.2 | DsbB | 2 $e^-$ | 1 (0.1) |
| 5.A.3 | PMO | Proton translocation | 7 (0.8) |
| 8.A.1 | MFP | Proteins, peptides, lipopolysaccharides, drugs, dyes, signalling molecules, heavy metal ions, etc. | 25 (2.7) |
| 8.A.3 | MPA1 | Complex polysaccharides | 3 (0.3) |
| 8.A.4 | MPA2 | Complex polysaccharides | 1 (0.1) |
| 8.A.7 | EI | Sugars | 1 (0.1) |
| 8.A.8 | HPr | Sugars | 1 (0.1) |
| 9.A.2 | MerTP | $Hg^{2+}$ (uptake) | 3 (0.3) |
| 9.A.8 | FeoB | $Fe^{2+}$ (uptake) | 1 (0.1) |
| 9.A.10 | OFeT | $Fe^{2+}$ (uptake) | 3 (0.3) |
| 9.A.17 | PbrT | Lead resistance | 2 (0.2) |
| 9.A.21 | ComC | DNA, proteins | 1 (0.1) |

Copyright © 2005 John Wiley & Sons, Ltd. 

**Table 4.** Continued

| Family | Abbreviation | Typical substrates | No. of members (%) |
|---|---|---|---|
| 9.B.3 | MPE | Lipid-linked murein precursors, such as NAG–NAM–pentapeptide pyrophosphoryl undecaprenol (lipid II) | 2 (0.2) |
| 9.B.4 | PET | Unknown | 4 (0.4) |
| 9.B.10 | MarC | Multiple antibiotic resistance | 1 (0.1) |
| 9.B.14 | HEP | Heme | 2 (0.2) |
| 9.B.17 | FAT | Fatty acyl CoA ligases (fatty acyl CoA synthases), carnitine CoA ligases, and putative transporters | 30 (3.2) |
| 9.B.20 | MgtC | $Mg^{2+}$ | 2 (0.2) |
| 9.B.22 | PerM | Unknown | 1 (0.1) |
| 9.B.24 | TEGT | Glucose (and fructose?) uptake or metabolism, cell death | 1 (0.1) |
| 9.B.26 | PC-terminal fragment (7) | Unknown | 1 (0.1) |
| 9.B.30 | Hly III | Unknown | 1 (0.1) |
| 9.B.32 | VGP | Polysaccharides | 2 (0.2) |
| 9.B.37 | HCC | Ions? | 3 (0.3) |
| 9.B.40 | DotA/TraY | Unknown | 1 (0.1) |
| 9.B.42 | ExeAB | Secretin | 1 (0.1) |
| 9.B.43 | YedZ | Unknown | 1 (0.1) |
| 9.B.45 | YnfA | Unknown | 1 (0.1) |
| 9.B.53 | UIT6 | Unknown | 1 (0.1) |
| | Unclassified | Unknown | 1 (0.1) |
| Total | | | 932 (100) |

The RND superfamily of export pumps is well represented, with 30 members. Of these, over half (17) in subfamily 1 are predicted to function in heavy metal efflux. Another nine (in subfamily 2) probably export drugs and other hydrophobic and amphipathic substances. The RND proteins of Rme have been compared to those from other bacteria recently (Nies, 2003). The two SecDF system components (subfamily 4), facilitate protein secretion via the general secretory pathway (Sec; 3.A.5). Lipid (subfamily 5) and pigment (subfamily 8) exporters may also be present.

Another well-represented superfamily encoded within the genome of Rme is the drug/metabolite transporter (DMT) superfamily, with 18 members within four of the families of this superfamily. Most of these transporters (families 1, 2 and 3) probably function in drug and metabolite efflux, but one (family 7) may be a sugar uptake permease.

A single putative 2-keto-3-deoxygluconate uptake permease was identified. Additionally, one member of the CitMHS (citrate uptake) family and one member of the LctP lactate uptake family were found. One system may export $Ca^{2+}$ (CaCA family) while another may import phosphate (PiT family). A surprise was the identification of a member of the ATP : ADP antiporter (AAA) family, because such transporters were previously predominantly identified in intracellular pathogenic organisms and rarely in other bacteria (until now in *Ralstonia eutropha* strain JMP134, *Pseudomonas fluorescens, Pirella, Rhodopirellula baltica* and *Magnetospirillum magnetotacticum*). However, what it could be doing in a free-living organism remains to be determined.

Five members of the SSS family most resemble characterized permeases for organoanions and cations as well as a putative nitrogen sensor. All of the five members of the DAACS family are predicted to transport dicarboxylates. These may include the two dicarboxylate amino acids, aspartate and glutamate. A putative CCS family member is also predicted to take up dicarboxylates. The four DASS family members probably serve similar functions but may also take up tricarboxylate compounds.

Both the CPA1 and CPA2 monovalent cation antiporter families are represented, with one and six members, respectively. CPA1 family members are predicted to be $Na^+ : H^+$ antiporters, while CPA2 family members may be $K^+$ efflux systems. Three NCS2 nucleobase/nucleoside uptake systems and

Copyright © 2005 John Wiley & Sons, Ltd.

two Amt ammonia/ammonium transporters were identified.

Two putative arsenite exporters (one of the ArsB-type and one of the Acr3-type) were found. Four potential chromate resistance (CHR) pumps and five putative sulphate uptake permeases (SulP) may be involved in chromate and sulphate metabolism, respectively. The CHR and SulP porters may be functionally related, since chromate is a sulphate analogue.

Six constituents of the tripartite TRAP-T family (2.A.56) may comprise three distinct systems for dicarboxylate uptake. However, studies indicate that members of this family may transport substrates of diverse structure, rendering substrate identification difficult. Only two TRAP-T receptors but at least three large and one small integral membrane constituents of these systems were identified. Because of rapid sequence divergence of the small integral membrane constituents, some of these proteins may have been missed. This situation can be contrasted with the superficially similar tripartite TTT family (2.A.80), where 74 potential constituents were found. Interestingly, about five proved to resemble the large and 11 the small integral membrane constituents of these systems, while 58 proved to be homologous to TTT family receptors. The occurrence of multiple probable receptors for TTT family systems in some bacteria has been noted before (Antoine *et al.*, 2003).

Several additional families of transporters are probably involved in nutrient uptake (BenE, OPT and AAE) and metabolite efflux (AEC, LysE, RhtB and LIV-E). All of these are concerned with transport of peptides, amino acids and their derivatives. The largest of these families is the RhtB family, with 11 members. Additionally, constituents of a TonB–ExbBD system, which probably functions primarily to energize transport across the outer membrane by a proton electrophoretic mechanism, were identified.

A complete twin arginine targeting (TatABC) system, as well as a single Oxa1 homologue, is encoded within the genome of Rme. These two independently acting systems function in the secretion of a subset of extracellular proteins and in the insertion of integral membrane proteins, including redox enzymes, respectively (Yen *et al.*, 2002). Genome analyses of the leader sequences of potential secretory proteins should reveal which are substrates of the Tat system and which are exported via the Sec system.

### Primary active transporters

The vast majority of protein constituents of primary active transporters encoded within the Rme genome are members of the ABC superfamily; 213 proteins in Rme belong to this superfamily, 181 putative uptake system proteins and 32 putative efflux system proteins. Most ABC systems consist minimally of two membrane protein (M) and two ATP hydrolysing cytoplasmic protein (C) subunits which may be fused in various combinations. Consequently, the basic unit of an ABC transporter may be encoded by a single gene or up to four distinct genes. Additionally, extracytoplasmic receptors are associated with all uptake systems, and there may be several of these per system. Therefore, it is not possible to estimate accurately the number of intact ABC transporters present. The problem is exacerbated by the fact that the constituents of ABC systems are often encoded within multiple, non-adjacent operons.

Table 4 summarizes the family associations of the various ABC transporter constituents. The ratio of sugar uptake system constituents (CUT1 + CUT2) to amino acid plus peptide uptake systems (PAAT + HAAT + PepT) is 15 : 52 or about 1 : 4. This fact, together with the corresponding analyses of secondary carriers discussed above, reveals the much greater dependency of Rme on amino acid metabolism than carbohydrate metabolism (see also Table 2). Values for numbers of sugar and amino acid transporter constituents can be compared with the total number of organic and inorganic anion and cation uptake transporter constituents (about 20 of each). ABC-type efflux systems are concerned with the export of drugs (10), complex carbohydrates (5), heme (6), proteins (5) and heavy metals (7) (Tables 3 and 4).

Rme has a single multicomponent F-type ATPase for the interconversion of chemical and chemiosmotic energy. It also possesses a dozen paralogous cation transporting P-type ATPases. Three of them have been characterized in detail (Borremans *et al.*, 2001; Legatzki *et al.*, 2003a) and all of them have been compared to P-type ATPases from other bacteria (Nies, 2003). Recently, the ongoing annotation work (**http://genome.ornl.gov/microbial/rmet/**)

Copyright © 2005 John Wiley & Sons, Ltd. 

identified another P-type ATPase (ZP_00 273 867) that was not included here.

A complete multicomponent general protein secretory (Sec) system (TC #3.A.5) was found in Rme, and this system undoubtedly serves as the primary protein export system for transport of proteins from the cytoplasm to the periplasm (Cao and Saier, 2003). However, Rme also has types II (MTB), III and IV macromolecular export systems. The first of these functions exclusively to export proteins across the outer membrane, but the latter two transport their substrates across both membranes. Type IV systems may also function in conjugation, and, in plant pathogens, in DNA export to the host cell. Additional potential DNA translocation proteins of the DNA-T, S-DNA-T and FphE families were also identified (Table 3). However, assignment of their specific functional roles must await experimental studies.

The $Na^+$ transporting carboxylate decarboxylases (TC #3.B.1) are multicomponent systems where the $\beta$-subunit catalyses $Na^+$ export in response to cytoplasm substrate decarboxylation catalysed by the $\alpha$-subunit. These systems minimally require the presence of $\alpha$-, $\beta$- and $\gamma$-subunits (Dimroth *et al*., 2001). One such system may be present in Rme.

### Proton pumping electron carriers

Rme has a single member of each of the three proton- or sodium-translocating electron transfer complexes of the NADH dehydrogenase (NDH), quinol : cytochrome *c* reductase (QCR) and cytochrome oxidase (COX) families. It also has at least two multicomponent transhydrogenases (PTH family). Rme therefore has a complete electron transfer chain for oxidizing NADH, using molecular oxygen as electron acceptor. All four electron carrier complexes cited above have the potential to generate an ion motive force as a primary source of energy. These coupled systems probably function together under aerobic conditions. Other transmembrane electron flow systems that can influence cellular energetics (class 5A and 5B) were also identified.

### Group translocators

The complete phosphoenolpyuvate–sugar phosphotransferase system (PTS; TC #4.A) is present in Rme. It includes, however, just one mannose (Man)-type PTS permease (Zhang *et al*., 2003). Only one Enzymes I and one HPr were identified. It is clear that Rme possesses a minimal PTS, in agreement with the earlier conclusion, based on secondary and primary active transporter analyses, that Rme is not strongly dependent on sugar metabolism as a source of energy.

### Poorly-defined transporters

Among the poorly characterized permeases of TC class 9.A, Rme has systems that probably transport heavy metal ions: mercury, iron, lead and magnesium. Several putative permeases of TC class 9.B were also identified (Table 3), but their functions are not known.

### Perspectives and conclusions

We have analysed transporters in the heavy metal-resistant organism, *R. metallidurans* (Rme). This organism possesses several $\alpha$-type channel proteins. Some are concerned specifically with monovalent or divalent inorganic cation or anion transport, but several non-specific stress response channels also appear to be present. Rme also has a huge repertoire of outer membrane $\beta$-barrel porins involved in transport of small molecules as well as macromolecules across the outer membrane. Many (e.g. OMRs) are probably specific for uptake, while others (e.g. OMFs) mediate efflux.

Regarding secondary carriers for sugars, Rme seems to have a very limited repertoire of such systems relative to most other sequenced Gram-negative bacteria, such as *E. coli* and other enteric bacteria. Thus, Rme has only one MFS carbohydrate transporter in the sugar porter family. It has no putative glycoside transporters of the GPH family (TC #2.A.2). It does have a putative 2-keto-3-deoxygluconate transporter of the KDGT family, and it has a few ABC uptake transporters specific for monosaccharides and small oligosaccharides of the CUT1 and CUT2 subfamilies, as well as a complete phosphotransferase system. Rme may only transport hexoses via the one PTS permease identified.

The capacity of Rme to transport carboxylic acids and their derivatives as sources of carbon appears to be fairly extensive. Thus, several families of secondary mono- and dicarboxylate carriers

Copyright © 2005 John Wiley & Sons, Ltd. 

(MFS, DAACS, DASS and TRAP-T) were identified. It also possesses members of the tricarboxylate transporting CitMHS, CCS and TTT families (Winnen *et al*., 2003). ABC-type carboxylate transporters were also found. Thus, the results point to a strong respiratory-type metabolism, with greater dependency on exogenous organic acids than carbohydrates.

Our genome analyses revealed several transporters that are probably specific for amino acids, peptides and their derivatives. Thus, for the uptake of amino acids, three families of secondary carriers were represented [MFS (MHS), APC and SSS], while members of two ABC families with this specificity (PAAT and HAAT) were found. For the uptake of peptides, two potential families of secondary carriers (OPT, MPE) and one ABC family (PepT) were represented. Finally, for amino acid efflux, members of five potential families were identified (DMT, AEC, LysE, RhtB and LIV-E). It seems clear that the transport and metabolism of amino acids and their derivatives is of considerable importance to the lifestyle of Rme.

Our analyses also revealed a large number of potential drug/hydrophobe/amphiphile export systems. Many of these belong to the DHA1, -2 and -3 families of the MFS. While a few of these efflux pumps may be involved in sugar export (Table 3; Saier, 2000), it is possible that some export amino acids and their derivatives, particularly those of a hydrophobic nature. It should be noted, however, that this has not yet been established for any member of the three MFS DHA families.

Other families, including transporters that probably export hydrophobic substances, include the HAE1 family in the RND superfamily, and the DME family of the DMT superfamily. At least some of these are probably concerned with drug export. Members of the MATE family within the MOP superfamily and several putative drug exporters of the ABC superfamily may serve similar functions. All of these families are represented in Rme. The diversity of substrates exported by these systems has yet to be studied.

As noted in Table 2 and further exemplified in Tables 3 and 4, over 220 transporters in Rme are probably concerned with inorganic ion transport. The following families are represented (see Table 3): (1) for monovalent cations: VIC, CytB, MscL, MscS, CPA1, CPA2, Amt, KUP, F-ATPase, P-ATPase and four proton-translocating electron carriers (NDH, PTH, QCR and COX); (2) for di- or trivalent cations: MIT, NNP(MFS), CDF, ZIP, RND, CaCA, NiCoT, FeCT(ABC), MZT(ABC), P-ATPase and MgtC; and (3) for anions: MFS, Pit, ArsB, DASS, CHR, SulP, PNaS, ACR3, SulT(ABC), PhoT(ABC), MolT(ABC) and NitT(ABC).

Inspection of Table 3 reveals possible transporters for a variety of additional interesting metabolites, such as organic anions (benzoate, phenylacetate, cyanate, phosphonates, sulphonates). Transporters specific for osmolytes, both purine and pyrimidine bases and nucleosides, quaternary ammonium compounds and possibly nucleotides (ADP/ATP in the AAA family), were also identified.

An extensive repertoire of macromolecular exporters was found. Protein secretion and membrane protein insertion systems include the Sec, Tat, Oxa1 and types I–IV systems. Complex carbohydrates can probably be exported via MOP, ABC and VGP family transporters. Possible lipid exporters of the RND superfamily have been identified, and several MFS and ABC systems may similarly catalyse lipid ‘flip-flop’, which is equivalent to export from the inner leaflet of the cytoplasm membrane bilayer to the outer leaflet. Some of these transporters may also export lipids from the inner membrane to the outer membrane.

Finally, several of the identified transporters could not be assigned even a tentative function. It should also be kept in mind that transporters that belong to functionally uncharacterized families may not be included in the TC system and therefore may not be identified using the computer approaches used here. Although our studies have revealed a disproportionate number of transporters concerned with inorganic ion transport, particularly with heavy metal resistance, and while these studies clearly point to the dominant types of metabolic activity upon which Rme depends for energy, it is clear that we are only at the beginning of an understanding of the scope of molecular transport processes in *Ralstonia metallidurans*.

### Acknowledgements

This work was supported by NIH Grant No. GM64368. We thank the Joint Genomic Institute (JGI) for permitting us use of the preliminary genomic sequence of *R. metallidurans*, without which this study would not have been

Copyright © 2005 John Wiley & Sons, Ltd. 

possible. We thank Mary Beth Hiller for her assistance in the preparation of this manuscript.

### References

Andres Y, Thouand G, Boualam M, Mergeay M. 2000. Factors influencing the biosorption of gadolinium by microorganisms and its mobilization from sand. *Appl Microbiol Biotechnol* **54**: 262–267.

Antoine R, Jacob-Dubuisson F, Drobecq H, *et al.* 2003. Overrepresentation of a gene family encoding extracytoplasmic solute receptors in *Bordetella*. *J Bacteriol* **185**: 1470–1474.

Anton A, Weltrowski A, Haney JH, *et al.* 2004. Characteristics of zinc transport by two bacterial cation diffusion facilitators from *Ralstonia metallidurans* and *Escherichia coli*. *J Bacteriol* **186**: 7499–7507.

Arispe N, De Maio A. 2000. ATP and ADP modulate a cation channel formed by Hsc70 in acidic phospholipid membranes. *J Biol Chem* **275**: 30 839–30 843.

Borremans B, Hobman JL, Provoost A, Brown NL, van Der Lelie D. 2001. Cloning and functional analysis of the pbr lead resistance determinant of *Ralstonia metallidurans* CH34. *J Bacteriol* **183**: 5651–5658.

Boucher C, Genin S, Arlat M. 2001. Current concepts on the pathogenicity of phytopathogenic bacteria. *C R Acad Sci III* **324**: 915–922.

Busch W, Saier MH Jr. 2002. The transporter classification (TC) system, 2002. *CRC Crit Rev Biochem Mol Biol* **37**: 287–337.

Cao TB, Saier MH Jr. 2003. The general protein secretory pathway: phylogenetic analyses leading to evolutionary conclusions. *Biochim Biophys Acta* **1609**: 115–125.

Degen O, Eitinger T. 2002. Substrate specificity of nickel/cobalt permeases: insights from mutants altered in transmembrane domains I and II. *J Bacteriol* **184**: 3569–3577.

Dimroth P, Jockel P, Schmid M. 2001. Coupling mechanism of the oxaloacetate decarboxylase $Na^+$ pump. *Biochim Biophys Acta* **1505**: 1–14.

Eberz G, Eitinger T, Friedrich B. 1989. Genetic determinants of a nickel-specific transport system are part of the plasmid-encoded hydrogenase gene cluster in *Alcaligenes eutrophus*. *J Bacteriol* **171**: 1340–1345.

Eitinger T, Friedrich B. 1991. Cloning, nucleotide sequence, and heterologous expression of a high-affinity nickel transport gene from *Alcaligenes eutrophus*. *J Biol Chem* **266**: 3222–3227.

Eitinger T, Friedrich B. 1994. A topological model for the high-affinity nickel transporter of *Alcaligenes eutrophus*. *Mol Microbiol* **12**: 1025–1032.

Eitinger T, Wolfram L, Degen O, Anthon C. 1997. A $Ni^{2+}$ binding motif is the basis of high affinity transport of the *Alcaligenes eutrophus* nickel permease. *J Biol Chem* **272**: 17 139–17 144.

Goris J, De Vos P, Coenye T, *et al.* 2001. Classification of metal-resistant bacteria from industrial biotopes as *Ralstonia campinensis* sp. nov., *Ralstonia metallidurans* sp. nov. and *Ralstonia basilensis*. *Int J Syst Evol Microbiol* **51**: 1773–1782.

Juhnke S, Peitzsch N, Hubener N, Grosse C, Nies DH. 2002. New genes involved in chromate resistance in *Ralstonia metallidurans* strain CH34. *Arch Microbiol* **179**: 15–25.

Kimball R, Saier MH Jr. 2002. Voltage-gated $H^+$ channels associated with human phagocyte superoxide-generating NADPH oxidases: sequence comparisons, structural predictions, and phylogenetic analyses. *Mol Membr Biol* **19**: 137–147.

Krogh A, Larsson B, von Heijne G, Sonnhammer E. 2001. Predicting transmembrane protein topology with a hidden Markov model. Application to complete genomes. *J Mol Biol* **305**: 567–580.

Legatzki A, Grass G, Anton A, Rensing C, Nies DH. 2003a. Interplay of the Czc system and two P-type ATPases in conferring metal resistance to *Ralstonia metallidurans*. *J Bacteriol* **185**: 4354–4361.

Legatzki A, Franke S, Lucke S, *et al.* 2003b. First step towards a quantitative model describing Czc-mediated heavy metal resistance in *Ralstonia metallidurans*. *Biodegradation* **14**: 153–168.

Mergeay M, Houba C, Gerits J. 1978. Extrachromosomal inheritance controlling resistance to cadmium, cobalt and zinc ions: evidence from curing in a *Pseudomonas*. *Arch Int Physiol Biochem* **86**: 440–441.

Mergeay M, Monchy S, Vallaeys T, *et al.* 2003. *Ralstonia metallidurans*, a bacterium specifically adapted to toxic metals: towards a catalogue of metal-responsive genes. *FEMS Microbiol Rev* **27**: 385–410.

Mergeay M, Nies D, Schlegel HG, *et al.* 1985. *Alcaligenes eutrophus* CH34 is a facultative chemolithotroph with plasmid-bound resistance to heavy metals. *J Bacteriol* **162**: 328–334.

Mouz S, Merlin C, Springael D, Toussaint A. 1999. A GntR-like negative regulator of the biphenyl degradation genes of the transposon Tn4371. *Mol Gen Genet* **262**: 790–799.

Munkelt D, Grass G, Nies DH. 2004. The chromosomally encoded CDF proteins DmeF and FieF are transporters of broad specificity and DmeF is necessary for cobalt detoxification through the CzcCBA efflux system. *J Bacteriol* **186**: 8036–8043.

Nies DH. 2003. Efflux-mediated heavy metal resistance in prokaryotes. *FEMS Microbiol Rev* **27**: 313–339.

Nottebrock D, Meyer U, Kramer R, Morbach S. 2003. Molecular and biochemical characterization of mechanosensitive channels in *Corynebacterium glutamicum*. *FEMS Microbiol Lett* **218**: 305–309.

Paulsen IT, Nguyen L, Sliwinski MK, Rabus R, Saier MH Jr. 2000. Microbial genome analyses: comparative transport capabilities in eighteen prokaryotes. *J Mol Biol* **301**: 75–100.

Pivetti CD, Yen M-R, Miller S, *et al.* 2003. Two families of prokaryotic mechanosensitive channel proteins. *Microbiol Mol Biol Rev* **67**: 66–85.

Roux M, Sarret G, Pignot-Paintrand I, Fontecave M, Coves J. 2001. Mobilization of selenite by *Ralstonia metallidurans* CH34. *Appl Environ Microbiol* **67**: 769–773.

Saier MH Jr. 2000. A functional–phylogenetic classification system for transmembrane solute transporters. *Microbiol Mol Biol Rev* **64**: 354–411.

Saier MH Jr. 2003. Answering fundamental questions in biology with bioinformatics. *ASM News* **69**: 175–181.

Salanoubat M, Genin S, Artiguenave F, *et al.* 2002. Genome sequence of the plant pathogen *Ralstonia solanacearum*. *Nature* **415**: 497–502.

Copyright © 2005 John Wiley & Sons, Ltd. 

Silver S. 2003. Bacterial silver resistance: molecular biology and uses and misuses of silver compounds. *FEMS Microbiol Rev* **27**: 341–353.

Springael D, Ryngaert A, Merlin C, Toussaint A, Mergeay M. 2001. Occurrence of Tn4371-related mobile elements and sequences in (chloro)biphenyl-degrading bacteria. *Appl Environ Microbiol* **67**: 42–50.

Taghavi S, Mergeay M, van der Lelie D. 1997. Genetic and physical maps of the *Alcaligenes eutrophus* CH34 megaplasmid pMOL28 and its derivative pMOL50 obtained after temperature-induced mutagenesis and mortality. *Plasmid* **37**: 22–34.

Toussaint A, Merlin C, Monchy S, *et al*. 2003. The biphenyl- and 4-chlorobiphenyl-catabolic transposon Tn4371, a member of a new family of genomic islands related to IncP and Ti plasmids. *Appl Environ Microbiol* **69**: 4837–4845.

Tran CV, Yang NM, Saier MH Jr. 2003. TC-DB: An architecture for membrane transport protein analysis. Proceedings of the 2nd International IEEE Computer Society Computational Systems Bioinformatic Conference (CSB 2003), Stanford, CA; 658.

Vandamme P, Coenye T. 2004. Taxonomy of the genus *Cupriavidus*: a tale of lost and found. *Int J Syst Evol Microbiol* **54**: 2285–2289.

Vaneechoutte M, Kämpfer P, De Baere T, *et al*. 2004. *Wautersia* gen. nov., a novel genus accommodating the phylogenetic lineage including *Ralstonia eutropha* and related species, and proposal of *Ralstonia* [*Pseudomonas*] *syzygii* (Roberts, *et al*., 1990) comb. nov. *Int J Syst Evol Microbiol* **54**: 317–327.

Winnen B, Hvorup RN, Saier MH Jr. 2003. The tripartite tricarboxylate transporter (TTT) family. *Res Microbiol* **154**: 457–465.

Wolfram L, Eitinger T, Friedrich B. 1991. Construction and properties of a triprotein containing the high-affinity nickel transporter of *Alcaligenes eutrophus*. *FEBS Lett* **283**: 109–112.

Wolfram L, Friedrich B, Eitinger T. 1995. The *Alcaligenes eutrophus* protein HoxN mediates nickel transport in *Escherichia coli*. *J Bacteriol* **177**: 1840–1843.

Yen M-R, Tseng YH, Nguyen EH, Wu LF, Saier MH Jr. 2002. Sequence and phylogenetic analyses of the twin arginine targeting (Tat) protein export system. *Arch Microbiol* **177**: 441–450.

Zhai Y, Saier MH Jr. 2001. A web-based program (WHAT) for the simultaneous prediction of hydropathy, amphipathicity, secondary structure and transmembrane topology for a single protein sequence. *J Mol Microbiol Biotechnol* **3**: 501–502.

Zhang Z, Aboulwafa M, Smith M, Saier MH Jr. 2003. The ascorbate transporter of *Escherichia coli*. *J Bacteriol* **185**: 2243–2250.

Copyright © 2005 John Wiley & Sons, Ltd. 
